# USP19 modulates cancer cell migration and invasion and acts as a novel prognostic marker in patients with early breast cancer

**DOI:** 10.1038/s41389-021-00318-x

**Published:** 2021-03-13

**Authors:** Fabiana Alejandra Rossi, Juliana Haydeé Enriqué Steinberg, Ezequiel Hernán Calvo Roitberg, Molishree Umesh Joshi, Ahwan Pandey, Martin Carlos Abba, Beatrice Dufrusine, Simonetta Buglioni, Vincenzo De Laurenzi, Gianluca Sala, Rossano Lattanzio, Joaquín Maximiliano Espinosa, Mario Rossi

**Affiliations:** 1 Instituto de Investigaciones en Medicina Traslacional (IIMT) - CONICET, Universidad Austral, Pilar, Buenos Aires Argentina; 2 Instituto de Investigación en Biomedicina de Buenos Aires (IBioBA-CONICET-MPSP), Buenos Aires, Argentina; 3 Functional Genomics Facility, University of Colorado School of Medicine, Aurora, CO USA; 4 Peter MacCallum Cancer Centre, Melbourne, VIC Australia; 5 Centro de Investigaciones Inmunológicas Básicas y Aplicadas, Facultad de Ciencias Médicas – Universidad Nacional de La Plata, La Plata, Buenos Aires Argentina; 6 Department of Innovative Technologies in Medicine & Dentistry, Center for Advanced Studies and Technology (CAST), University of Chieti-Pescara, Chieti, Italy; 7 Advanced Diagnostics and Technological Innovation Department, Regina Elena Cancer Institute, Rome, Italy; 8 Linda Crnic Institute for Down Syndrome, University of Colorado School of Medicine, Aurora, CO USA; 9 Department of Pharmacology, University of Colorado School of Medicine, Aurora, CO USA

**Keywords:** Breast cancer, Functional genomics, Ubiquitylation

## Abstract

Tumor cell dissemination in cancer patients is associated with a significant reduction in their survival and quality of life. The ubiquitination pathway plays a fundamental role in the maintenance of protein homeostasis both in normal and stressed conditions and its dysregulation has been associated with malignant transformation and invasive potential of tumor cells, thus highlighting its value as a potential therapeutic target. In order to identify novel molecular targets of tumor cell migration and invasion we performed a genetic screen with an shRNA library against ubiquitination pathway-related genes. To this end, we set up a protocol to specifically enrich positive migration regulator candidates. We identified the deubiquitinase USP19 and demonstrated that its silencing reduces the migratory and invasive potential of highly invasive breast cancer cell lines. We extended our investigation in vivo and confirmed that mice injected with USP19 depleted cells display increased tumor-free survival, as well as a delay in the onset of the tumor formation and a significant reduction in the appearance of metastatic foci, indicating that tumor cell invasion and dissemination is impaired. In contrast, overexpression of USP19 increased cell invasiveness both in vitro and in vivo, further validating our findings. More importantly, we demonstrated that USP19 catalytic activity is important for the control of tumor cell migration and invasion, and that its molecular mechanism of action involves LRP6, a Wnt co-receptor. Finally, we showed that USP19 overexpression is a surrogate prognostic marker of distant relapse in patients with early breast cancer. Altogether, these findings demonstrate that USP19 might represent a novel therapeutic target in breast cancer.

## Introduction

Cell migration plays a crucial role in a wide variety of physiological processes^1^. Its activation is highly regulated both spatially and temporarily, contributing to the maintenance of tissue and cellular homeostasis^1,2^. Therefore, it is not surprising that when deregulated, migration is associated with the development and progress of multiple pathologies, including cancer^2–4^.

Alteration or exacerbation of malignant tumor cell migration and dissemination is the principal cause of death due to solid tumors^5^.

In addition, it was observed that decreasing the migratory capabilities of tumor cells can restore and increase the susceptibility to chemotherapeutic treatments^6,7^. Consequently, targeting genes that regulate cell motility could be beneficial in the treatment of highly aggressive cancers^8–10^.

Cell motility is a complex process that requires post-translational regulation of a wide variety of proteins. Ubiquitination is an important form of protein post-translational modification that consists in the conjugation of ubiquitin polypeptides to target proteins^11,12^, and is responsible for regulating different processes^13,14^. The reversion or modification of poly-ubiquitin chains is carried out by deubiquitinating enzymes (DUBs)^15^.

To identify novel molecular targets within the ubiquitination pathway that positively regulate migration we conducted a loss-of-function genetic screen using an epithelial cell line derived from human triple-negative breast cancer (MDAMB231) infected with a pooled shRNA interference library. This type of cancer is associated with aggressive behavior and an overall poor prognosis^16^.

From our screen, we identified the Ubiquitin-specific protease 19 (USP19) as a candidate gene associated with the regulation of cell migration. USP19 presents different isoforms, some of them have a cytoplasmic localization while others have a transmembrane domain that serves as anchorage to the endoplasmic reticulum^17,18^. This DUB is associated with protein quality control and cellular homeostasis^17–21^. In particular, it has been demonstrated that USP19 regulates LRP6 stability, a co-receptor of the Wnt signaling cascade^22^. Aberrant activation of this pathway and LRP6 polymorphisms and overexpression have been associated with susceptibility to the development of different cancers, including breast cancer^23–27^.

To validate USP19 function as a positive regulator of migration and invasion, we performed a series of in vitro and in vivo experiments analyzing USP19’s role in colonization and tumor formation. In addition, we showed that USP19 overexpression is associated with distant relapse in patients diagnosed with early breast cancer. Collectively, our data suggest that USP19 plays a crucial role in breast cancer cell dissemination, and we provide novel evidence that it can be a prognostic marker and attractive candidate for the development of new therapeutic strategies.

## Materials and methods

### Cell lines and cell culture

Cell lines were obtained from the ATCC and cultured in Dulbecco’s modified Eagle’s medium (DMEM) (Gibco) supplemented with 10% fetal bovine serum (FBS) (Natocor, Córdoba, Argentina), 50 U/ml penicillin-streptomycin, and 200 μM L-glutamine at 37 °C and 5% CO_2_ in a humidified incubator. ATCC uses morphology, karyotyping, and PCR-based approaches to confirm the identity of human cell lines. Mycoplasm contamination was evaluated monthly by PCR, and cell lines were cultured less than three months.

### shRNA screening and plasmid transfections

A pool of plasmids encoding 1885 shRNAs targeting 407 different genes related to the ubiquitination pathway in the pLKO.1 backbone produced by The RNAi Consortium (TRC, Sigma-Aldrich, St. Louis, MO) were obtained from the University of Colorado.

For single shRNA transduction, TRCN51715 and TRCN51716 (USP19 shRNA# 1 and 2, respectively), TRCN33406, and TRCN33408 (LRP6 shRNA# 1 and 2, respectively), and SHC001 PLKO.1 vectors were used.

For overexpression experiments, transfections were performed using Lipofectamine 2000 reagent (Invitrogen, Carlsbad, CA). GFP-tagged wild type and catalytically dead mutant (C506S) USP19 plasmids were a gift of Dr. Urbé (University of Liverpool, UK), and GFP-tagged ΔTM USP19 plasmid was obtained by generating a premature stop by mutagenesis PCR from wild-type USP19 vector.

### Transwell migration assay

After starvation for 24 h (0.1% FBS), 5 × 10^4^ cells were added to the top chamber of 24-well transwells (BD Bioscience, Bedford, MA, Cat#353097), and 10% FBS assay medium was added to the bottom chambers and incubated for 24 h. After non-migratory cell removal, membranes were fixed, stained with 4′,6-diamidino-2-phenylindole and imaged using a Zeiss Axio Observer Z1 Inverted Epi-fluorescence microscope.

For the screen, 6.6 × 10^5^ starved MDAMB231 cells were plated onto 6-well transwell chambers (BD Bioscience, Bedford, MA, Cat# 353093). After a 24-hour incubation, the non-migratory cells were collected, propagated, and allowed to re-migrate for enrichment purposes. Cells from 8 transwells were combined per cycle to ensure a >700 library coverage. Simultaneously, migration in each cycle was determined in 24-well plates as described before.

### Quantitative PCR

Total RNA was extracted using TRIzol reagent (Invitrogen, Thermo Fisher Scientific) and cDNA synthesis was carried out using M-MLV reverse transcriptase in the presence of RNasin RNase**-**inhibitor (Promega) and an oligo(dT) primer (Invitrogen).

Quantitative real-time PCR was carried out using the FastStart Essential DNA Green Master kit (Roche) at an annealing temperature of 60 °C for 35 cycles, and a CFX96 PCR Detection System (Biorad). Expression was calculated by the comparative CT (ΔCT) method with GAPDH for normalization.

### Western blot analysis

Cells were lysed in lysis buffer (50 mM Tris-HCl pH 7.4, 250 mM NaCl, 25 mM NaF, 2 mM EDTA, 0.1% Triton-X, with protease inhibitors mix (Complete ULTRA, Roche), 1 mM 1,4-DTT, 1 μM NaOV, 10 nM okadeic acid), and protein concentrations were determined using the BCA assay Kit (Pierce). Equal amounts of protein were separated by 8–12% SDS-PAGE and transferred to PVDF membranes (Millipore-Merck). Membranes were incubated with primary antibodies: rabbit anti-USP19 (Bethyl Cat#A301-587A), mouse anti-tubulin and mouse anti-GFP (Santa Cruz Biotechnology, Cat#sc-398103, and Cat#sc-9996 B2, respectively), mouse anti-β-actin and rabbit anti-LRP6 (Cell Signaling, Cat#3700 and Cat#2560 C5C7, respectively) and HRP-conjugated secondary antibodies: anti-rabbit and anti-mouse (GE Healthcare Cat#NA934 and Cat#NA931, respectively), and then detected using an ECL SuperSignal West Femto and West Pico detection kit (Pierce).

### Wound-healing assays

Confluent monolayers were starved for 24 h (0.1% FBS) and a single scratch was created using a micropipette tip. Cells were washed and incubated with 3% FBS medium at 37 °C to enable migration.

### Agar invasion assay

The procedure was performed as previously^28–30^ with minor modifications, included in the Supplementary Materials.

### Noble agar assay

This experiment was performed as previously^31^ with minor modifications, described in the Supplementary Materials.

### Matrigel three-dimensional cell culture

Experiments were carried out based on experimental settings described before^32–35^. A detailed description is included in the Supplementary Materials.

### Mouse tumorigenesis and metastasis models

NOD SCID mice were originally purchased from Jackson Laboratories (Bar Harbor, ME, USA), and bred in IBioBA’s animal facility under a pathogen-free environment. For all experiments, 7/8-week-old mice were used in accordance with protocols approved by the Institutional Board on Animal Research and Care Committee (CICUAL, Experimental Protocol #63, 22.nov.2016), School of Exact and Natural Sciences, University of Buenos Aires. 4 weeks after birth, mice from each sex were randomly divided at a density no more than 5 animals/cage. At the time of injection, cages for the different treatments were arbitrary selected. Sample size was calculated using G*Power (version 3.1.9.6; Heinrich Heine University Düsseldorf, Germany). The following design specifications were taken into account: *α* = 0.05; (1-ß) = 0.8; effect size *ƒ* = 0.4.

For in vivo mouse tumor studies, 5 × 10^5^ cells in 100 μl of PBS were subcutaneously injected in the mammary fat pads of female mice. Tumors were measured every 3 days and tumor volumes were calculated using the formula: Volume = ½ (width^2^ × length). Area Under Curve analysis was performed using measurements from mice alive at the end of the experiment.

For the experimental metastasis assay, 1 × 10^6^ cells in 200 μl of PBS were injected in the lateral tail vein of male mice. Lungs were harvested 60 days post-injection, fixed in buffered formalin and then stored in 70% ethanol until use for DNA quantification (as described before ref. ^36^) or paraffin embedding, or insufflated with a 15% India Ink solution and counterstained with Fekete’s solution for macrometastasis exposure and imaging.

### In silico analysis of USP19 mRNA expression among the TCGA-BRCA dataset

Pre-processed USP19 expression levels among 800 primary breast carcinomas with intrinsic subtype data and their integrated pathway activities (pathway activity - z score of 1387 constituent PARADIGM pathways) were obtained from the TCGA Breast Cancer (BRCA) dataset at UCSC Xena browser (http://xena.ucsc.edu/). The PARADIGM algorithm integrates pathway, expression, and copy number data to infer activation of pathway features within a superimposed pathway network structure extracted from NCI-PID, BioCarta, and Reactome^37^.

Briefly, Luminal A/B primary breast cancer group (*n* = 600) was divided into low (*n* = 77) or high (*n* = 209) USP19 expression levels according to the StepMiner one-step algorithm (http://genedesk.ucsd.edu/home/public/StepMiner/). These two groups were then compared at their integrated pathway activities to identify the most relevant signaling pathways associated with USP19 expression using the SAM test (*p* < 0.01; Fold Change > 1.5) with MultiExperiment Viewer Software (MeV 4.9).

### Patients immunohistochemistry

Patients inclusion criteria are described in the Supplementary Materials. Tissue microarrays (TMA) were constructed by punching 2-mm-diameter cores from invasive breast carcinoma areas, as previously described^38^. TMA sections were incubated overnight with the rabbit anti-USP19 polyclonal antibody (LifeSpan, Cat#LS-C353286) or the anti-E-cadherin mouse monoclonal antibody (clone HECD-1, Zymed Laboratories Inc., San Francisco, CA), after applying the MW antigen retrieval technique at 750 W for 10 min in 10 mM Sodium Citrate Buffer (pH 6.0).

The immunohistochemical analysis was carried out by two pathologists (R.L., S.B.) by agreement, with both blinded to the clinicopathological information. USP19 expression in patients’ samples was reported as the percent of cells with positive cytoplasmatic staining, and dichotomized (high vs. low, Supp. Fig. 1) according to the ROC analysis. The optimal cut-off parameter for USP19 positive expression was 50%. E-cadherin positivity was defined as a membrane-associated, linear pattern of immunoreactivity which decorated the cell membrane entirely.

The immunohistochemical results for the estrogen receptor (ER), progesterone receptor (PR), Ki67, and HER2 status were obtained from the patient hospital records.

Disease-free survival (DFS) was defined as the interval from surgery to the first of the following events: tumor relapse at local or distant sites. Distant relapse-free survival (DRFS) was defined as the time from surgery to the occurrence of distant relapse.

### Statistical analysis

Results are presented as Box-and-whisker plots with median interquartile ranges plus minimum to maximum. *n* indicates the number of independent replicates. The one-way ANOVA with Dunnett’s multiple-comparison test as well as non-parametric Kruskal–Wallis and Dunn’s Tests were used to compare treatments to their corresponding control, and adjusted p-values are indicated. *P*-value differences of <0.05 were considered statistically significant. GraphPad Prism and SPSS (version 15.0, Chicago, IL) statistical software were used.

Pearson’s *χ*^2^ or Fisher’s exact tests were used to assess the relations between the tumor USP19 protein expression and the patient clinicopathological parameters. The Log-Rank (Mantel-Cox) test was used to analyze differences between the survival curves, and Cox’s proportional hazard model was used to evaluate the association of USP19 expression with survival time, using covariates (tumor size, grade, and ER, PR, Ki-67, HER2, and USP19 status).

## Results

### Migration-based screen to identify ubiquitination-pathway genes with novel regulatory functions

**Fig. 1:**
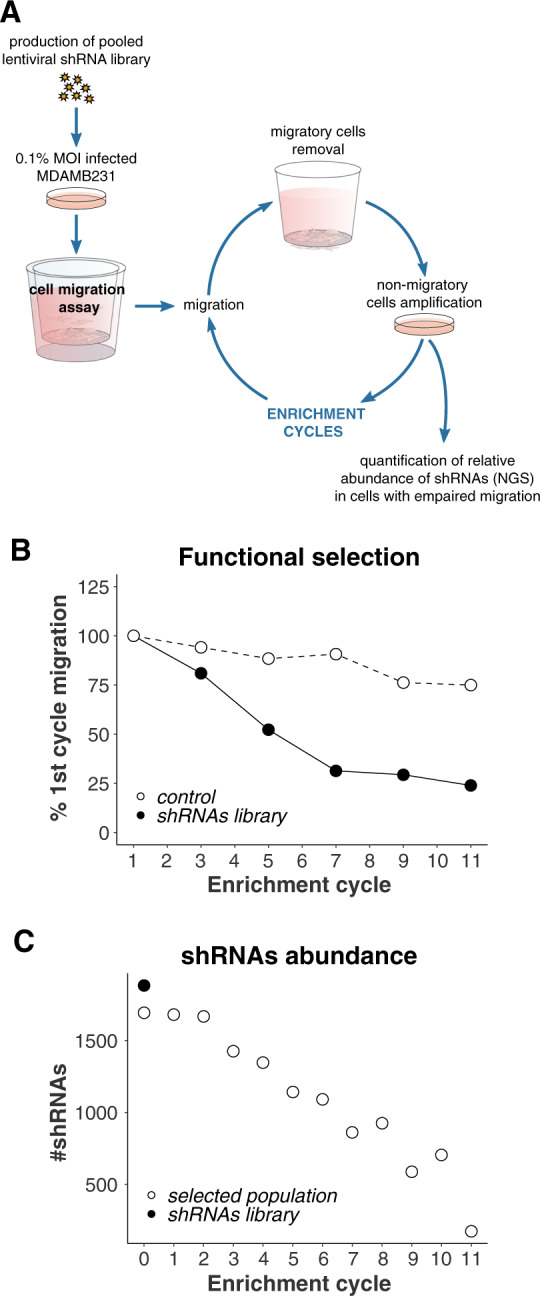
shRNA-based selection of positive regulators of cell migration. **A** Overview of the selection procedure. The production and infection of a ubiquitination-related lentiviral shRNA library are described in Methods. Two weeks after lentiviral infection and selection, MDAMB231 cells were seeded onto transwell inserts and allowed to migrate across the porous membrane for 24 h in order to select cells with a decreased migration phenotype. Migrating cells were removed and non-migrating cells were collected from the inserts upper compartment and amplified. Cells were then reseeded onto transwell culture inserts for a subsequent cycle of selection; this procedure was repeated until cells lost 80% of their initial migratory potential. After every cycle of selection, the relative abundance of the different shRNAs was evaluated using Next-Generation Sequencing. **B** Transwell assay was used every other enrichment cycle to determine the percentage of migratory cells and monitor the selection process. **C** shRNAs’ abundance was estimated after each selection cycle.

In order to identify novel positive regulators of cell migration within the ubiquitination pathway, we performed an shRNA-based functional selection screen (Fig. 1A). A pooled recombinant lentiviral shRNA library targeting over 400 human ubiquitination-related genes was stably transduced into breast cancer cells. The functional selection consisted in placing the mixed population into the upper compartment of a transwell unit and allowing migration through the perforated membrane to the lower compartment. Cells that exhibited reduced migration were isolated and amplified. We performed subsequent enrichment cycles until cells lost about 80% of their initial migratory potential (Fig. 1B). After every enrichment cycle, we evaluated shRNAs relative abundance in the cell population by PCR amplification and quantitative sequencing from genomic DNA. As shown in Fig. 1C, as enrichment cycles increased, we observed a marked reduction in the number of shRNAs, suggesting that the selection process was efficient. As a control, we used an empty vector-transduced cell line.

### Selection of candidate genes

**Fig. 2:**
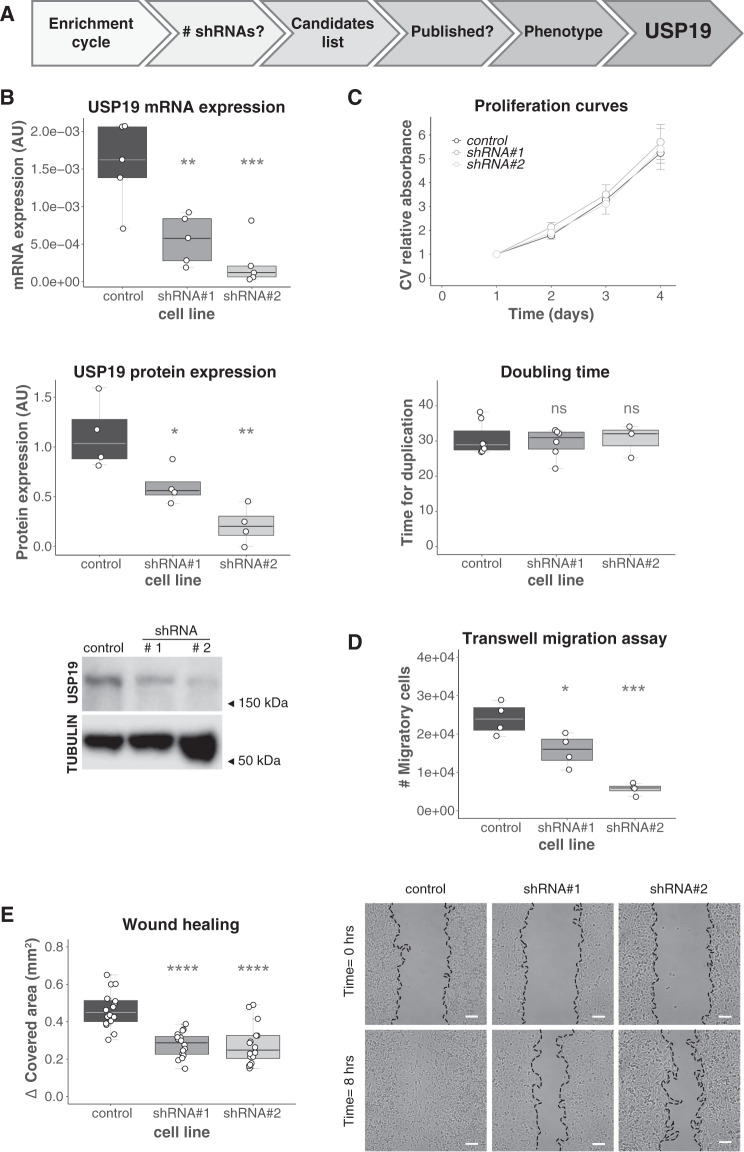
Validation and characterization of USP19 as a regulator of cell migration. **A** Workflow used to select a candidate regulatory gene. MDAMB231 cells were stably transduced with control empty vector shRNA (control) or two different shRNAs (#1 & #2) targeting USP19. **B** Efficiencies of shRNA-mediated knockdown were confirmed by RT-PCR (top, *n* = 5, one-way ANOVA, Dunnett’s multiple comparison test. shRNA#1 *p* = 0.0329 and shRNA#2 *p* = 0.0013) and Western Blotting (middle and bottom, *n* = 4, one-way ANOVA, Dunnett’s multiple comparison test. shRNA#1 *p* = 0.0227 and shRNA#2 *p* = 0.0006). (**C**) Crystal violet (CV) staining was used to determine cell growth over time. Cells were seeded onto wells and allowed to attach. At the indicated time points, cells were fixed and then stained at the end of the experiment. The graph on the top shows the mean relative CV absorbance every 24 h. Doubling time was calculated for control and USP19 silenced cell lines on the bottom (*n* ≥ 3, Kruskal–Wallis, Dunn’s multiple comparison test. shRNA#1 *p* > 0.9999 and shRNA#2 *p* > 0.9999). The migratory potential was evaluated by two different experiments. **D** Transwell assay: After 24 h of incubation, USP19-depleted cells were stained for microscopic examination and the number of migratory cells was compared to control cells. The graph shows the number of migratory cells per transwell membrane (*n* = 4, one-way ANOVA, Dunnett’s multiple comparison test. shRNA#1 *p* = 0.0187 and shRNA#2 *p* = 0.0001). **E** Wound-healing assays: scratching with a pipette tip made a gap on a monolayer of the different cell cultures, and time-lapse imaging monitored the number of migrating cells across the border. After 8 h, cells exhibited different levels of migration. The graph on the left shows the gap covered area (mm^2^) after 8 h (*n* = 16, one-way ANOVA, Dunnett’s multiple comparison test. shRNA#1 *p* < 0.0001 and shRNA#2 *p* < 0.0001) and the images on the right show representative areas in a wound-healing experiment at the indicated time points. Scale bar = 100 μm.

After the selection process, we followed an analytical workflow to select candidate genes for further validation (Fig. 2A). In order to avoid false positives due to off-target effects, we discarded those genes for which only one shRNA targeting its sequence was found in the sequencing results. These criteria allowed us to identify 30 genes whose depletion altered migration. Half of these genes had already been associated with migration, invasion, metastasis or tumorigenesis, and served as a proof of principle for the efficacy and specificity of our screen (Supp. Fig. 2 and Supp. Table 1). Among the identified candidates, we focused our attention on the study of the deubiquitinase USP19.

### Validation of USP19 as a regulator of cell migration

In order to validate USP19 as a potential regulator of cell migration, we established stable MDAMB231 cell lines transduced individually with two different shRNAs targeting USP19 expression (named shRNA#1 and shRNA#2). Our results showed that both caused a significant reduction in USP19 mRNA and protein levels (Fig. 2B).

It is conceivable that shRNAs promoting cell proliferation may have also been enriched during the functional selection, as they provide cells an advantage during the in vitro amplification step. To discard this possibility, we performed proliferation curves of control and USP19-silenced cell lines. We observed no differences between the different cell lines doubling rates (Fig. 2C, Supp. Fig. 3), providing evidence for a direct role of USP19 in the control of cell migration.

Next, we used transwell migration and wound-healing assays to confirm the effect of USP19 depletion on cell motility. As shown in Fig. 2D and E, USP19 knockdown significantly decreased the migratory potential of cells relative to the control cell line. More detailed analysis on the wound-healing assay indicated that wound-edge cells speed and total displacement were significantly reduced in USP19 knockdown cells, and they presented a minor increase in persistence relative to control cells (Supp. Fig. 4). We also compared the effect on migration of USP19 silencing with USP10 silencing, one of the already published candidate genes obtained from our screen^39–41^. Our results indicate that knock down of both genes impair migration to a similar extent (Supp. Fig. 5).

Altogether, these experiments indicate that USP19 silencing affects cell migration in vitro. We further confirmed our findings using another highly invasive breast cancer cell line (Supp. Fig. 6).

### USP19 knockdown impairs invasion

Cell motility is often associated with increased tumor cell invasion and is a characteristic trait of aggressive tumor cells^42,43^. Therefore, we decided to investigate the effect of USP19 depletion on tumor cell invasion.

**Fig. 3:**
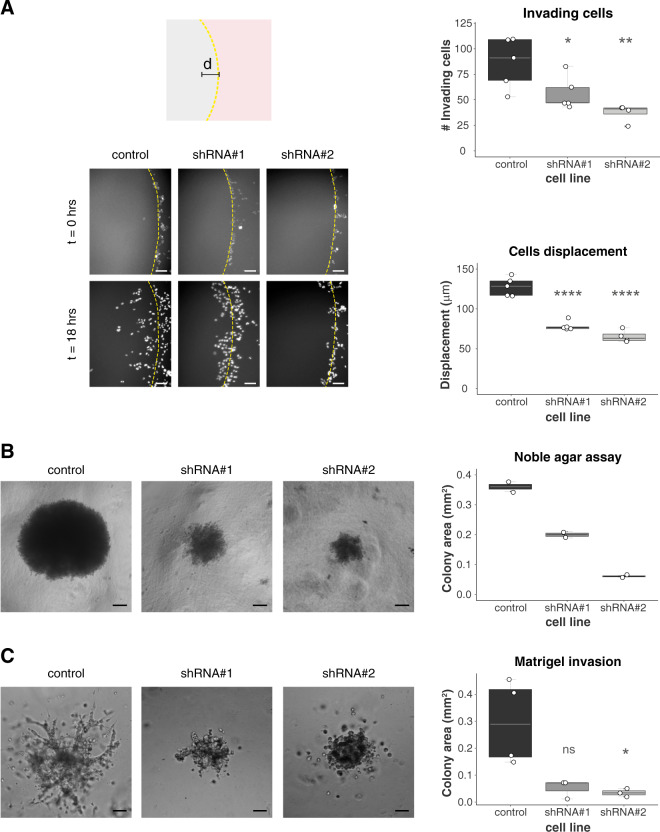
USP19 knockdown reduces cellular invasion. **A** Top left: Diagram of agar spot assay. MDAMB231 cells were seeded in wells (pink surface) with drops of solidified agar (gray sphere) and allowed to invade along the bottom surface under the agar. Pictures were taken along the edge (the edge is indicated by a dotted line); the displacement (d) is the extent of invasion under agar from the spot edge until the end of the experiment. Bottom Left: Representative area showing cell invasion into an agar spot at the indicate time points. Top right: Quantification of the mean number of invading cells per spot (*n* ≥ 4, one-way ANOVA, Dunnett’s multiple comparison test. shRNA#1 *p* = 0.0497 and shRNA#2 *p* = 0.0042) and bottom right: cells mean displacement after 18 h (*n* ≥ 4, one-way ANOVA, Dunnett’s multiple comparison test. shRNA#1 *p* < 0.0001 and shRNA#2 *p* < 0.0001). Scale bar = 100 μm. **B** Noble agar assay was used to study 3D culture proliferation and invasion. Left: Representative brightfield images obtained at 6 weeks in culture are shown. Scale bar = 150 μm. Right: Colony size was calculated at the end of the experiment (*n* = 2). **C** Left: Representative area showing cell invasion in a Matrigel 3D experiment after 5 days in culture. Right: Colony area was calculated at the end of the experiment (*n* ≥ 3, Kruskal–Wallis, Dunn’s multiple comparison test. shRNA#1 *p* = 0.1033 and shRNA#2 *p* = 0.0348). Scale bar = 150 μm.

To this end, we first analyzed the ability of cells to invade agar spots. Our results show that USP19 knockdown significantly reduced the number of invading cells as well as their total displacement, compared to the control cell line (Fig. 3A).

We next performed a 3D growth assay by seeding cells at low confluence into noble agar, an anchorage-independent matrix. After 6 weeks in culture, the control cell line formed bigger colonies compared to USP19-silenced cell lines (Fig. 3B), indicating that colonization, matrix invasion and anchorage-independent growth in these conditions is partially impaired in cells where USP19 expression is reduced.

Finally, we assessed growth and invasion into a reconstituted extracellular matrix that provides anchorage (Matrigel®). Cell lines expressing USP19 shRNAs showed colonies with a significantly smaller size than the control cell line (Fig. 3C), indicating that USP19 is required for an efficient invasion even when an anchorage is provided.

We further validated our results using another breast cancer cell line (Supp. Fig. 6).

Collectively, our results indicate that USP19 knockdown inhibits tumor cell invasion in vitro.

### USP19 overexpression enhances migration and invasion

We then analyzed the effect of USP19 overexpression in a poorly migratory and non-invasive breast cancer cell line (MCF7).

**Fig. 4:**
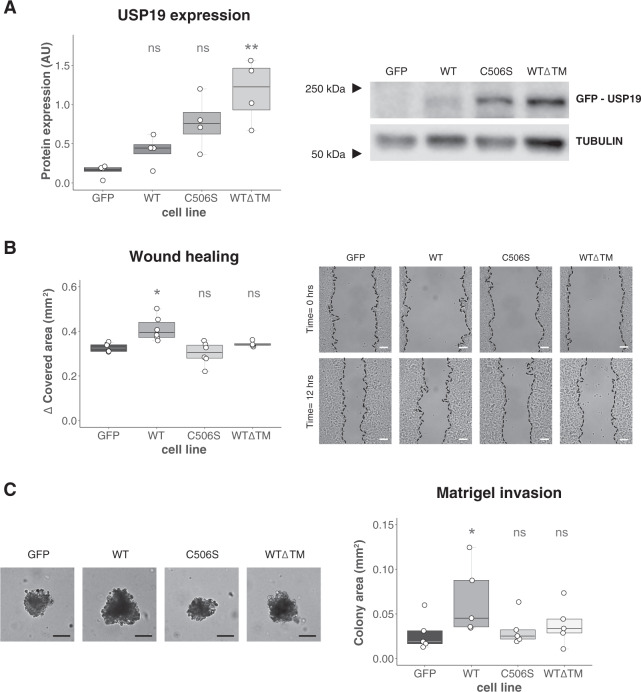
USP19 wild-type overexpression enhances migration and invasion in MCF7 cells. **A** Different constructs of USP19 were overexpressed in MCF7 cells, and their expression confirmed by Western Blotting (*n* = 4, Kruskal–Wallis, Dunn’s multiple comparison test. WT *p* > 0.9999, C506S *p* = 0.0777, and WTΔTM *p* = 0.0070). **B** The migratory potential was evaluated by wound-healing assay. Left: gap covered area (mm^2^) after 12 h (*n* ≥ 4, Kruskal–Wallis, Dunn’s multiple comparison test. WT *p* = 0.0107, C506S *p* > 0.9999, WTΔTM *p* > 0.9999) and right: representative areas in a wound-healing experiment at the indicated time points. Scale bar = 100 μm. **C** Matrigel invasion was assessed over a 30 days period. Left: Representative brightfield images obtained at the end of the experiment using a ×10 objective are shown; scale bar = 100 μm. Right: colony size was calculated (*n* ≥ 4, Kruskal–Wallis, Dunn’s multiple comparison test. WT *p* = 0.0485, C506S *p* > 0.9999, WTΔTM *p* > 0.9999).

For this purpose, we stably transfected MCF7 cells with a USP19 overexpressing plasmid (Fig. 4A), and then performed wound-healing assays. As shown in Fig. 4B, USP19 overexpression induced a significant increase in the gap covered area, compared to the control cell line. As a control, we overexpressed a catalytically mutant version of USP19^18,44–48^ and a mutant lacking USP19 transmembrane domain (Supp. Fig. 7). In contrast to USP19 wild type, we did not detect any substantial increase in migration in either of these mutants compared to the control cell line (Fig. 4B).

This result further supports the hypothesis that USP19 is a positive regulator of migration, and it provides evidence that this phenotype is dependent on its catalytic activity and on its subcellular localization.

Next, we analyzed the effect of USP19 overexpression on invasion and growth into a reconstituted extracellular matrix (Matrigel). We observed a significant increase in colony areas when comparing wild-type USP19 overexpressing cells to the control cell line (Fig. 4C). In accordance with our previous results, the USP19-dependent increase in invasion is also determined by its catalytic activity and presence of the transmembrane domain (Fig. 4C).

### USP19 regulates invasion in vivo

**Fig. 5:**
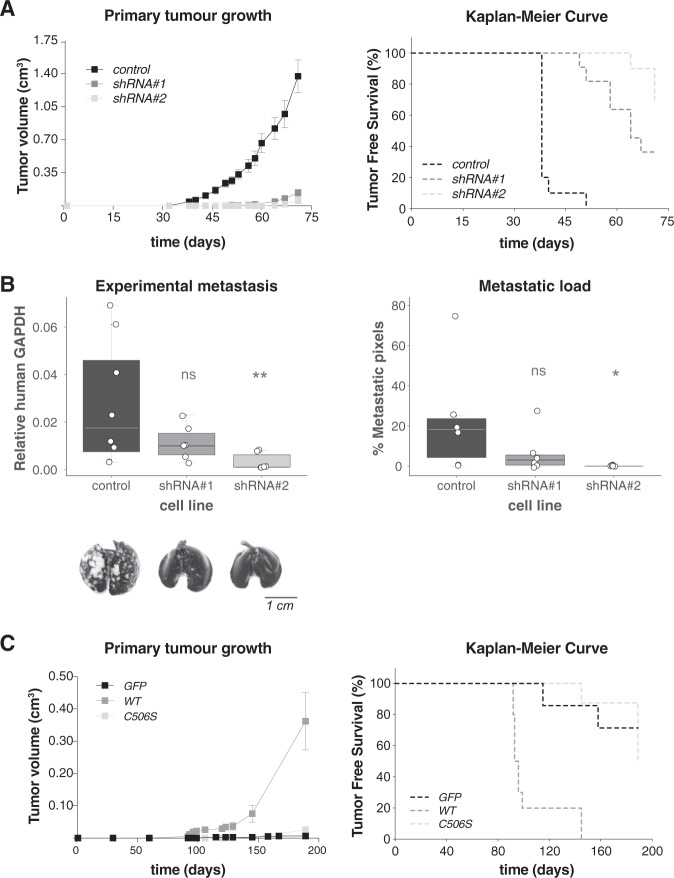
Analysis of USP19 expression relevance using mice models. (**A**) Downregulation of USP19 attenuates tumorigenicity in vivo: Control or USP19-silenced MDAMB231 cells were subcutaneously inoculated into the mammary fat pads of female NOD/SCID mice and tumor growth monitored every 2–3 days. Left: tumor volume was calculated at the indicated time points (results show mean value ± S.E.); right: Kaplan–Meier curves were built for tumor-free survival (TFS) over time (*n* ≥ 10, Log-Rank (Mantel-Cox) test, shRNA#1 *p* < 0.0001 and shRNA#2 *p* < 0.0001). **B** Silencing effects of USP19 on experimental metastasis assays: NOD/SCID male mice were inoculated with MDAMB231 USP19-silenced cells through tail vein injection and after 2 months, lungs were harvested. Top left: metastatic foci were estimated by qPCR human DNA quantification (*n* ≥ 6, Kruskal–Wallis, Dunn’s multiple comparison test. shRNA#1 *p* = 0.9950 and shRNA#2 *p* = 0.0032). Bottom left: representative lung images stained with Indian ink at the end of the experiment are shown. Scale bar = 1 cm. Right: metastatic load quantification was performed by evaluating lung Hematoxylin & Eosin-stained slides. We used a lesion-based analysis of percent of metastatic pixels to compare the differences in metastatic load produced on the lungs by the MDAMB231 cell lines (*n* = 6, Kruskal–Wallis, Dunn’s multiple comparison test. shRNA#1 *p* > 0.9999 and shRNA#2 *p* = 0.0299). **C** USP19 catalytic activity is needed for tumorigenicity in vivo: control, WT or C506S mutant versions of USP19 overexpressing MCF7 cells were subcutaneously inoculated into the mammary fat pads of female NOD/SCID mice. Left: tumor volume was calculated at the indicated time points (results show mean value ± S.E.); right: Kaplan–Meier curves were built for tumor-free survival (TFS) over time (*n* ≥ 7, Log-Rank (Mantel-Cox) test, WT *p* < 0.0001, and C506S *p* = 0.5307).

To further characterize USP19-dependent control of cell invasion in vivo, we first injected MDAMB231 control or USP19-silenced cells subcutaneously in the mammary fat pad of female mice. Tumor growth curves analysis indicated that those generated from control cells were significantly more volumetric than the ones originated from USP19-silenced cells (Fig. 5A, left and Supp. Fig. 8). Moreover, Kaplan–Meier curves for tumor-free survival indicated that cells expressing either of the shRNAs targeting USP19 generated fewer tumors (Fig. 5A, right and Supp. Table 2). In addition, we observed similar results using another breast cancer cell line (Supp. Fig. 9 and Supp. Table 2).

Second, we analyzed USP19’s role in the regulation of tumor cell lung colonization. For that purpose, we inoculated control or USP19-silenced MDAMB231 cells through tail vein injection and harvested the lungs two months later. As shown in Fig. 5B, USP19 depletion inhibits tumor foci formation in vivo, as evaluated by human DNA quantification (left) and metastatic load quantification in Hematoxylin & Eosin-stained lung sections (right, and Supp. Fig. 10). We observed the same trend when another breast cancer cell line was used (Supp. Fig. 9).

Last, we repeated the same type of tests using MCF7 cells in similar experimental conditions. We subcutaneously injected control cells or cells expressing either wild-type or catalytically mutant versions of USP19 in female mice.

In agreement with our in vitro experiments, wild-type USP19-expressing cells formed tumors in all injected mice, whereas mice injected with cells expressing the catalytic mutant did not show signs of tumor growth (Fig. 5C, Supp. Fig. 8 and Supp. Table 2). Since these cell lines showed no difference in proliferation rates in two dimensions (Supp. Fig. 11) and the fact that the MCF7 cell line does not usually form tumors unless an external estrogen source is supplied, this result highlights the importance of USP19 for tumor development and onset.

Altogether, we concluded that USP19 is important for in vivo colonization and tumor growth. In addition, our results indicate that USP19 catalytic activity and transmembrane domain are required for its stimulatory effect on cell motility.

### USP19 regulates LRP6 protein levels in breast cancer cells

**Fig. 6:**
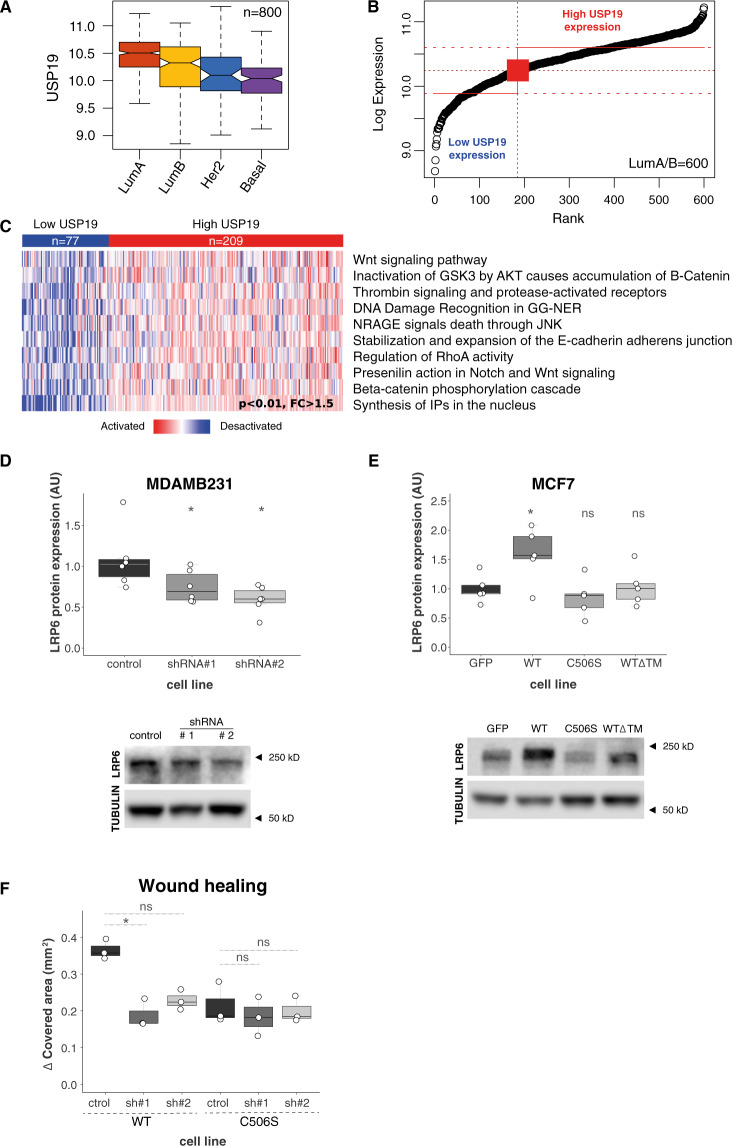
USP19 mechanism of action. An in silico study was performed in order to analyze the relationship between USP19 expression levels and different pathway activation status. **A** USP19 mRNA expression among primary breast carcinomas according to their intrinsic subtype. Expression analysis showed a consistent upregulation in luminal A and B subtypes compared with basal-like and Her2 subtypes. **B** Luminal A/B primary breast cancers divided into low (*n* = 77) or high (*n* = 209) USP19 mRNA expression levels. **C** Significantly activated pathways among Luminal A/B tumors with high USP19 mRNA expression (*n* ≥ 77, SAM test, *p* < 0.01). Western blotting was performed in order to analyze LRP6 protein expression in breast cancer cells upon USP19 genetic manipulation. **D** Top: Western blot quantification in control or USP19 silenced MDAMB231 cells (*n* = 6, one-way ANOVA, Dunnett’s multiple comparison test. shRNA#1 *p* = 0.0416 and shRNA#2 *p* = 0.0102), bottom: representative image of a blot. **E** Top: Western blot quantification in MCF7 cells overexpressing control or GFP-tagged USP19 constructs (*n* = 5, one-way ANOVA, Dunnett’s multiple comparison test. WT *p* = 0.0484, C506S *p* = 0.8469 and WTΔTM *p* = 0.9968), bottom: representative image of a blot. **F** Wound-healing assays were performed in order to analyze endogenous LRP6 silencing effects in MCF7 cells overexpressing WT or C506S mutant versions of USP19. Cells were stably transduced with a control vector (‘ctrol’, PLKO.1 empty vector), or shRNAs targeting LRP6 (sh#1 and sh#2). Scratching with a pipette tip made a gap on a monolayer of the different cell cultures, and time-lapse imaging monitored the number of migrating cells across the border. The graph shows the gap covered area (mm^2^) after 8 h (*n* = 3, Kruskal–Wallis and Dunn’s multiple comparison test for WT or C506S overexpressing MCF7 cell lines, analyzed separately. WT overexpressing MCF7 cell line: sh#1 *p* = 0.0341 and sh#2 *p* = 0.2021. C506S overexpressing MCF7 cell line: sh#1 *p* = 0.7422 and sh#2 *p* > 0.9999).

In order to study the putative mechanism of action responsible for USP19 migration and invasion regulation, we performed an in silico analysis on breast cancer mRNA expression using publicly available datasets. Our results revealed that high USP19 expression levels correlate with the activation of the Wnt pathway (Fig. 6A, B, and C).

This result was in concordance with previous observations by Perrody and collaborators, which demonstrated that USP19 stabilizes LRP6, a Wnt pathway coreceptor, and that this interaction affected downstream Wnt signaling^22^.

Based on these results, we analyzed LRP6 protein steady-state levels upon USP19 genetic manipulation. In accordance with Perrody et al., our results indicate that LRP6 protein levels decrease upon USP19 silencing in MDAMB231 (Fig. 6D) and increase in wild-type USP19-overexpressing MCF7 cells, but not in cells expressing catalytically dead or cytoplasmic mutant versions (Fig. 6E). This correlation was also observed when using another breast cancer cell line (Supp. Fig. 12).

In order to test the functional relation between USP19 and LRP6, we then analyzed the effect of LRP6 endogenous silencing in MCF7 cells overexpressing USP19. Our results indicated that wild-type USP19-induced increase in migration was reverted by LRP6 shRNAs stable expression (Fig. 6F).

Altogether, our results indicate that the axis USP19/LRP6, rather than the absolute level of expression of USP19 (Supp. Fig. 12), is key to regulate the migratory potential of breast cancer cells.

### Survival analysis of USP19 expression in early breast cancer patients

**Fig. 7:**
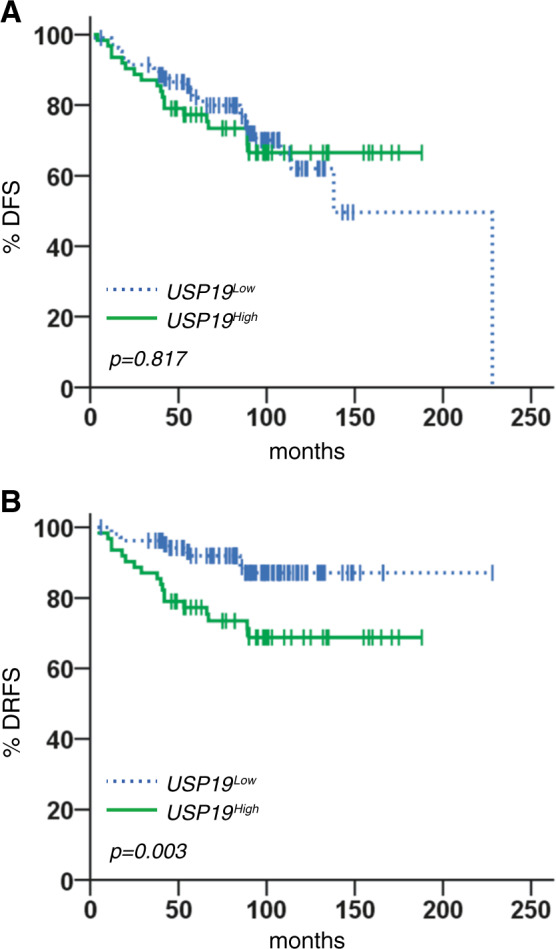
Prognostic value of USP19 protein expression in breast cancer patients. Kaplan–Meier estimates of disease-free survival (DFS) and distant relapse-free survival (DRFS) in patients with early breast cancer tumors, according to high (solid green lines) and low (dashed blue lines) expression of USP19. **A** 19 out of 62 patients (30.6%) harboring USP19^High^ tumors and 30 out of 106 patients (28.3%) with USP19^Low^ tumors had a disease relapse (*n* = 168, Log-Rank (Mantel-Cox) test, *p* = 0.817). **B** Distant metastases developed in 18 out of 62 (29.0%) and 11 out of 106 (10.4%) of patients with USP19^High^ and USP19^Low^ tumors, respectively (*n* = 168, Log-Rank (Mantel-Cox) test, *p* = 0.003).

Finally, we analyzed USP19 protein expression in a cohort study of early breast cancer patients with long-term follow-up. Kaplan–Meier plots showed that overexpression of USP19 was associated with a significantly lower frequency of DRFS, while no significant correlation with DFS was observed (Fig. 7A and B).

Multivariate analysis of DRFS, adjusted for other prognostic factors, revealed that USP19^High^ was an independent prognostic predictor of DRFS (Supp. Table 3).

Altogether these findings indicate that USP19 represents a new predictor of distant metastasis formation in early breast cancer patients.

## Discussion

Migration occurs in a wide variety of physiological conditions, and alterations in its regulation are associated with different pathologies, including cancer^3,4,49^. In this disease, mortality is associated primarily with tumor growth at secondary sites, and effective therapies to block the metastatic cascade are lacking^5^. In line with this reasoning, we chose to screen for genes that positively regulate motility within the ubiquitination pathway, as this cascade is currently emerging as an attractive therapeutic target in drug development^50–53^. Here we report the identification of USP19 as a positive regulator of migration in breast cancer. USP19 was initially characterized as a DUB predominantly localized in the cytosol in association with Hsp90 and other chaperones^21^. USP19 has been associated with the regulation of the half-life of several proteins that participate in different cellular processes^17,22,46,47,54–63^.

Our in vitro validation experiments showed that USP19 depletion did not affect cell proliferation in agreement with Lu et al.^64^, but directly inhibited cellular migration. In addition, we observed that USP19 knockdown impaired invasion (Figs. 2, 3, and Supp. Fig. 6) and also reduced anchorage-independent growth (Fig. 3).

To further confirm our results, we analyzed how USP19 overexpression affected migration and invasion, using a poorly migratory cell line. USP19 overexpression induced an increase in cellular migration, invasion, and growth in three-dimensional basement membrane cultures. These effects were dependent on USP19 subcellular-localization, and on the presence of a highly conserved cysteine at the catalytic site (Fig. 4).

Our in vivo studies using immunocompromised mice demonstrated that USP19 silencing decreased cell engraftment and tumor growth, as well as colonization into the lungs (Fig. 5A and Supp. Fig. 9B). On the contrary, overexpression of wild-type USP19, but not its catalytically deficient mutant version, promoted tumor growth (Fig. 5B). This is compatible with the requirement of USP19 catalytic activity for local invasion and growth in three dimensions, both in vitro and in vivo. In line with these results, we observed a marked increase in USP19 mRNA expression in cells growing in tumors compared to the same cells in culture dishes (Supp. Fig. 13). These results are compatible with a requirement for higher levels of USP19 to support three-dimensional invasion and growth, highlighting the possible existence of a specific regulation of USP19 in a context where cells need to invade.

Finally, a retrospective study conducted on human breast tumor samples indicated that high USP19 protein levels are associated with a high-risk for metastatic relapse in patients diagnosed with early breast cancer (Fig. 7).

Altogether these results provide evidence indicating that USP19 has great potential as a therapeutic target for drug development in breast cancer treatment.

To our knowledge, USP19 molecular mechanism of action in the regulation of migration and invasion in breast cancer cells was not investigated before.

Our results demonstrated that USP19 expression correlates with tumor growth and invasion. Supporting this, we analyzed E-cadherin protein expression levels in the samples of our retrospective study and observed an inverse correlation between USP19 and E-cadherin expression (Supp. Table 4). In agreement with our results, previous reports demonstrated that low E-cadherin expression holds a prognostic value as a predictor of poorer prognosis and more aggressive phenotypes in breast cancer^65,66^.

Moreover, we performed an in silico analysis on breast cancer mRNA expression publicly available datasets, which revealed that high USP19 expression levels correlate with the activation of the Wnt pathway (Fig. 6A, B and C). This is consistent with a recent work that showed that USP19 regulates LRP6 stability, a co-receptor of the Wnt signaling cascade^22^. Particularly in breast cancer, LRP6 is overexpressed in around a third of the patient samples, and its overexpression has been proposed as a distinctive feature of a specific class of breast cancer subtype^67^.

In this regard, our experiments show that LRP6 expression positively correlates with USP19 protein levels in breast cancer cells (Fig. 6D and E, and Supp. Fig. 12) and that overexpression of a catalytically dead mutant or a cytoplasmic version of USP19 has no effect on LRP6 (Fig. 6E), in concordance with the previous results^22^. Moreover, this molecular mechanism is specifically associated with USP19 modulation and it is not a general effect as a result of a change in migration, since downregulation of USP10 and its concomitant reduction in migration does not alter LRP6 protein levels (Supp. Fig. 5C). In all, our results are compatible with former experiments that demonstrated that LRP6 downregulation in breast cancer cell lines reduces their migratory and invasive potential^68^, as well as their ability to form colonies in soft agar^67^. More importantly, we show that endogenous LRP6 silencing abolishes USP19 overexpression-induced increase in migration (Fig. 6F). Consequently, our results indicate that the functional interaction between USP19 and LRP6 is key for the regulatory effect that USP19 exerts on the modulation of breast cancer cell migration and invasion.

Opposite to our findings, Hu and collaborators recently demonstrated that USP19 negatively regulates proliferation and migration in clear cell renal carcinoma^69^. In this type of cancer, the most relevant USP19 isoform is uc003cvz.3, which is mainly localized in the cytoplasm^70^. Based on our data showing that the control of cell migration in breast cancer cells is mainly exerted by the transmembrane USP19 isoform, it is plausible to assume that this difference could contribute to explain the divergent role that USP19 plays in these two different cellular contexts.

For all the reasons expressed before, we conclude that USP19 is relevant for the regulation of breast cancer cell dissemination and its expression levels correlate with a high risk of metastases development and could therefore represent a novel target for the management of breast cancer metastatic disease, in particular when LRP6 expression is relevant for determining patients’ outcome.

## Disclaimer

All authors have seen and approved the manuscript, and it hasn’t been accepted or published elsewhere.

## Supplementary information

Supplementary Material

Supplementary Table S1

Supplementary Table S4
