# Supplementary material for: USP19 modulates cancer cell migration and invasion and acts as a novel prognostic marker in patients with early breast cancer
Source: Oncogenesis. 2021 Mar 13;10(3):28. doi: 10.1038/s41389-021-00318-x (PMC7956144; doi:10.1038/s41389-021-00318-x)
Supplement: Supplementary file 1 — Supplementary Material [file 41389_2021_318_MOESM1_ESM.pdf]

SUPPLEMENTARY FIGURES

A B

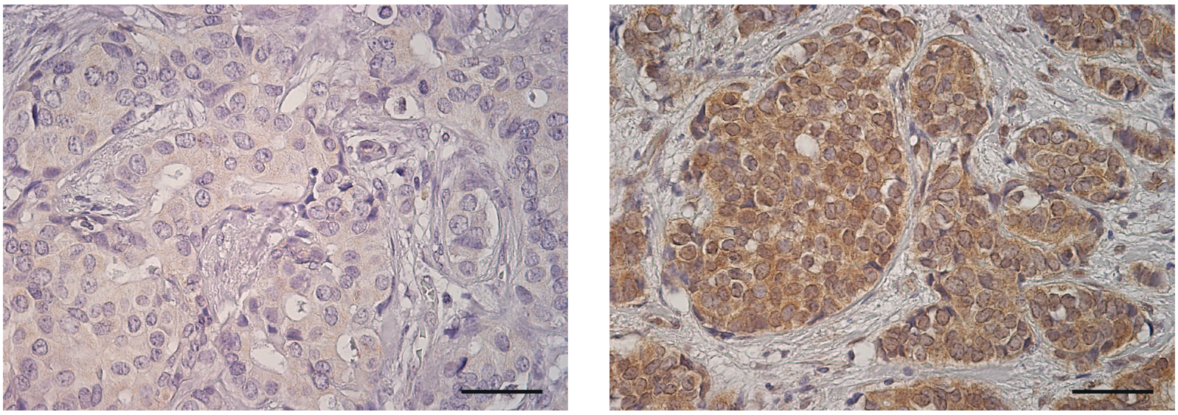

Figure S1l USP19 staining on FFPE breast cancer tissues.

Immunohistochemical staining: examples of (**A**) low and (**B**) high expression of USP19 in breast cancer. Scale bar: 200 µm.

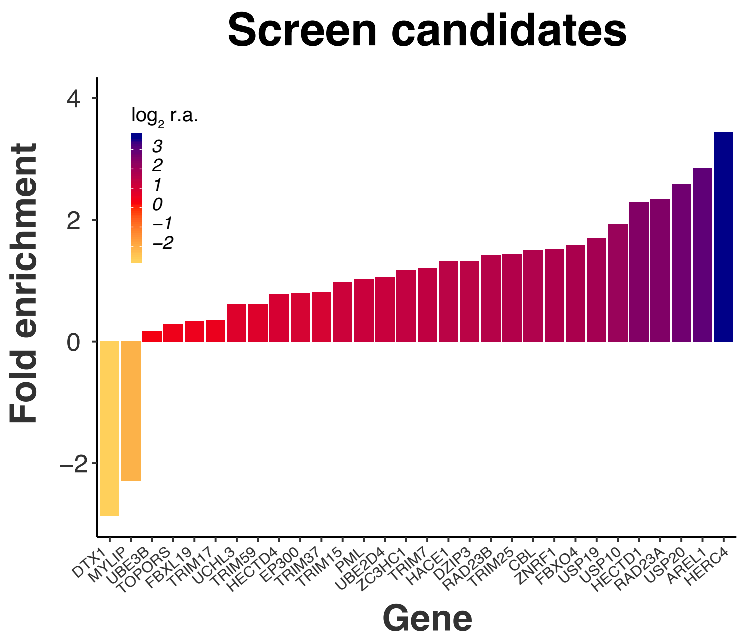

Figure S2I Candidate regulators of migration.

We performed a loss-of-function screen using shRNAs targeting ubiquitination pathway-related genes in MDAMB231 cells. We implemented a cyclic functional selection and focused our analysis in cells that presented impaired migration. By sequencing DNA of selected cells, we calculated the frequency of each shRNA relative to the whole population of shRNAs after every cycle of selection. We then calculated the fold enrichment of each shRNA as their relative abundance in the fourth cycle compared to their abundance in the non-selected population of cells. The graph shows the mean fold enrichment (log_2 of relative abundance [r.a.]) of at least two shRNAs targeting each candidate gene. Values above zero represent putative positive regulators of migration, and values below zero belong to shRNAs that were lost in the screen and could represent negative regulators of migration.

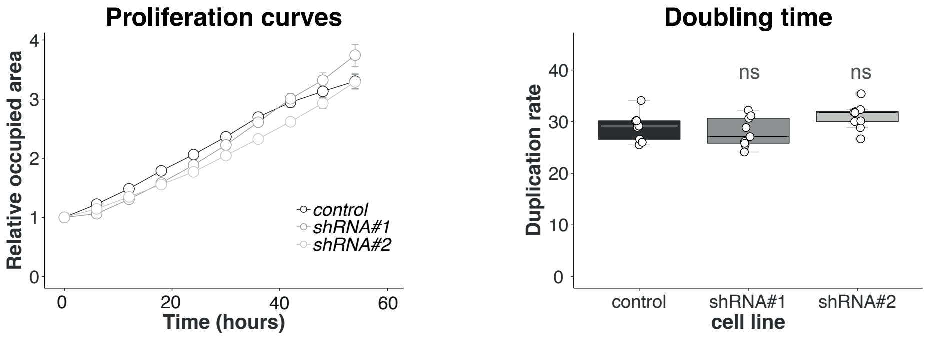

Figure S3l Doubling time of USP19 silenced MDAMB231 cells.

An area-based analysis of proliferation rate was used to determine cell growth over time. Cells were seeded onto wells, allowed to attach and imaged at the indicated time points. Left: relative mean occupied area at each time point ± S.E., and right: doubling time calculated for control and USP19 silenced cell lines (n= 9, one-way ANOVA, Dunnett's multiple comparison test. shRNA#1 p= 0.6284 and shRNA#2 p= 0.2123).

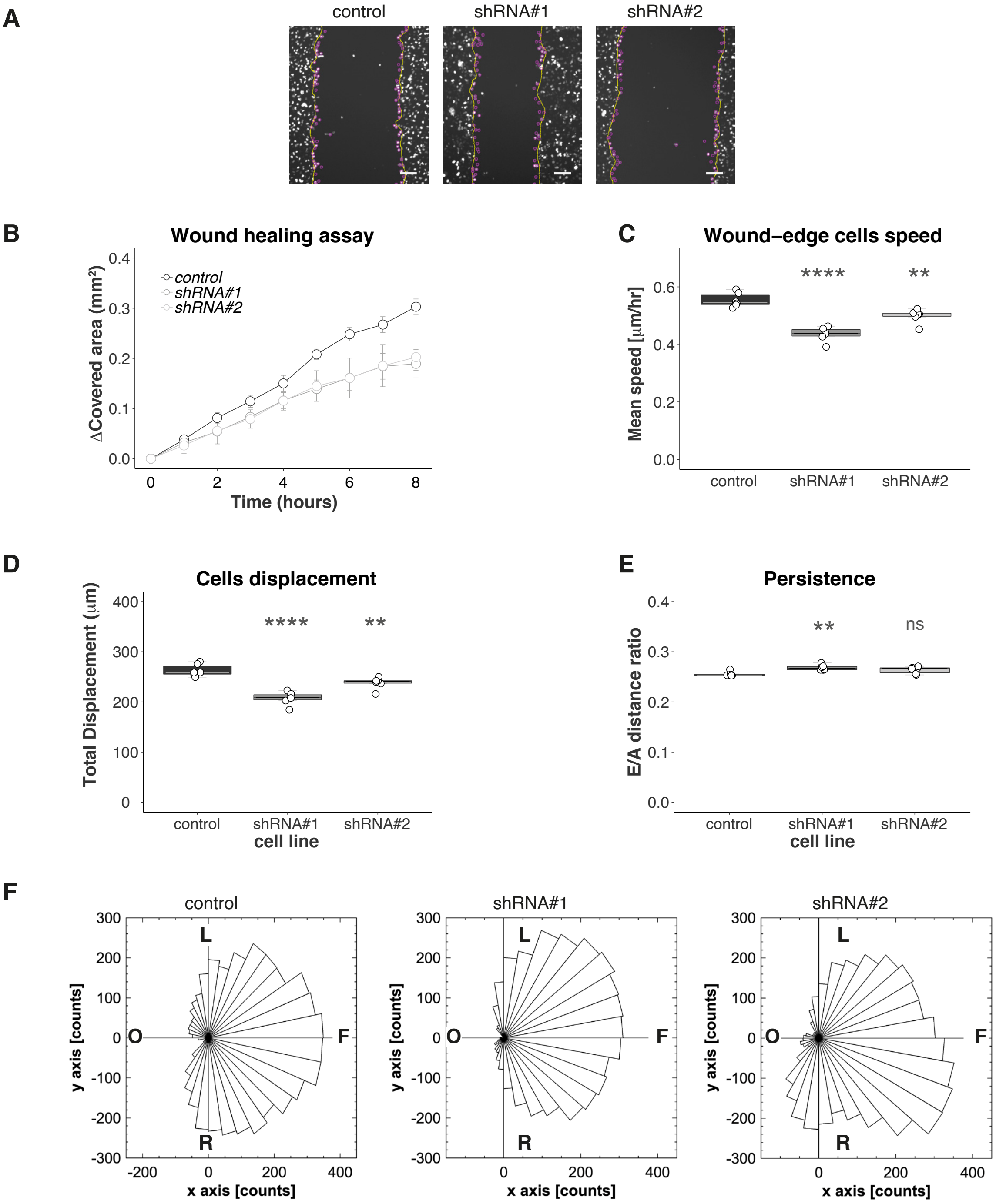

Figure S4I Detailed analysis on the wound healing assay in control or USP19 silenced MDAMB231 cells.

We generated MDAMB231 cells expressing GFP tagged-H2B and then stably transduced them with control vector or shRNAs # 1 and # 2 directed against USP19. For the wound healing assays, a scratch was made with a pipette tip on a monolayer of the different cell cultures, and time-lapse imaging monitored the number of migrating cells across the border. (**A**) Representative images of a wound healing assay at time= 0 hours. Cells circled in magenta were considered for the analysis. Data corresponding to circled cells in the gap area were manually excluded from the analysis. Data was collected from three independent experiments for each cell line. Scale bar= 100 µm. (**B**) The graph

shows the gap covered area (mm^2) at the indicated time. (**C**) Cells mean speed from each wound-edge was calculated throughout the length of the experiment. (n=6, one-way ANOVA, Dunnett's multiple comparison test. shRNA#1 p< 0.0001 and shRNA#2 p= 0.0028). (**D**) Mean accumulated track displacement (total displacement) for cells in each wound-edge was calculated until the end of the experiment (n=6, one-way ANOVA, Dunnett's multiple comparison test. shRNA#1 p< 0.0001 and shRNA#2 p= 0.0059). (**E**) Persistence was calculated as the Euclidean/Accumulated track displacement ratio for control or USP19 silenced MDAMB231 cells at each wound-edge. (n=6, one-way ANOVA, Dunnett's multiple comparison test. shRNA#1 p= 0.0003 and shRNA#2 p= 0.0527). (**F**) Rose diagrams representing the orientation of migration trajectories relative to cells position at time= 0 hours. Cells from the left wound-edge of three videos per cell line are shown, and each sector indicates the frequency of trajectory orientation. Control cell line (n= 131), shRNA#1 (n= 107) and shRNA#2 (n= 123). F: forward to direction of migration, L: left to direction of migration, O: opposite to direction of migration, R: right to direction of migration.

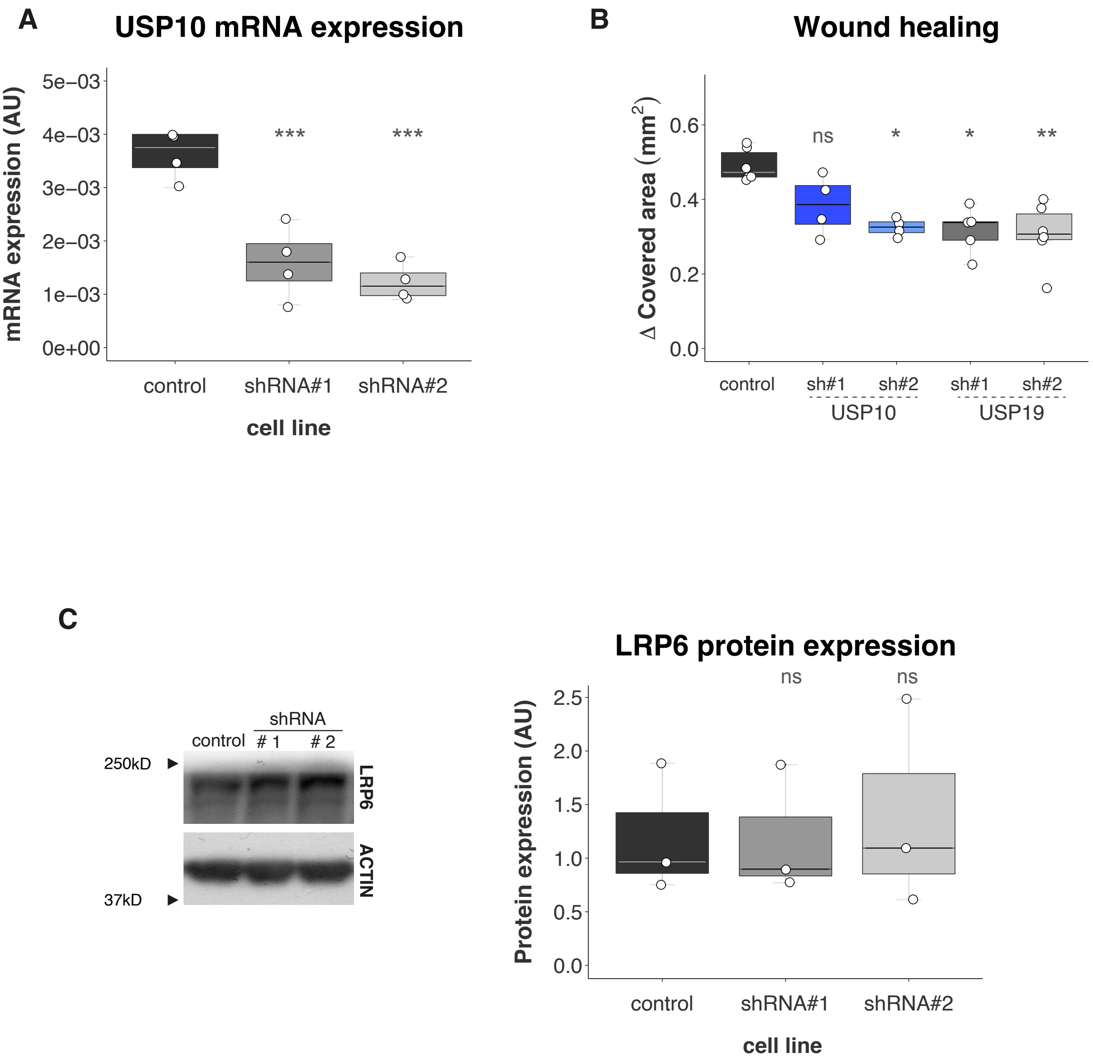

Figure S5I USP10 silencing effects in MDAMB231 cells migratory potential.

We generated USP10 silenced MDAMB231 cells and compared the effect on migration with USP19 depletion experiments. (**A**) Efficiencies of shRNA-mediated USP10 knockdown were confirmed by RT-PCR (n= 4, one-way ANOVA, Dunnett's multiple comparison test. shRNA#1 p= 0.0004 and shRNA#2 p= 0.0002, relative to mRNA expression in control cells). (**B**) Wound healing assays were used to analyze migration. The graph shows the gap covered area for control or cells stably expression shRNAs targeting USP10 or USP19 after 8 hours (n≥4 Kruskal-Wallis, Dunn's multiple comparison test. USP10 shRNA#1 p= 0.4576, USP10 shRNA#2 p= 0.0397, USP19 shRNA#1 p= 0.0141 and USP19 shRNA#2 p= 0.0060). (**C**) LRP6 protein expression was evaluated in control or USP10 silenced MDAMB231 cells. Left: representative image of a blot; right: LRP6 signal quantification (n=3, Kruskal-Wallis and Dunn's multiple comparison test. shRNA#1 p> 0.9999 and shRNA#2 p> 0.9999).

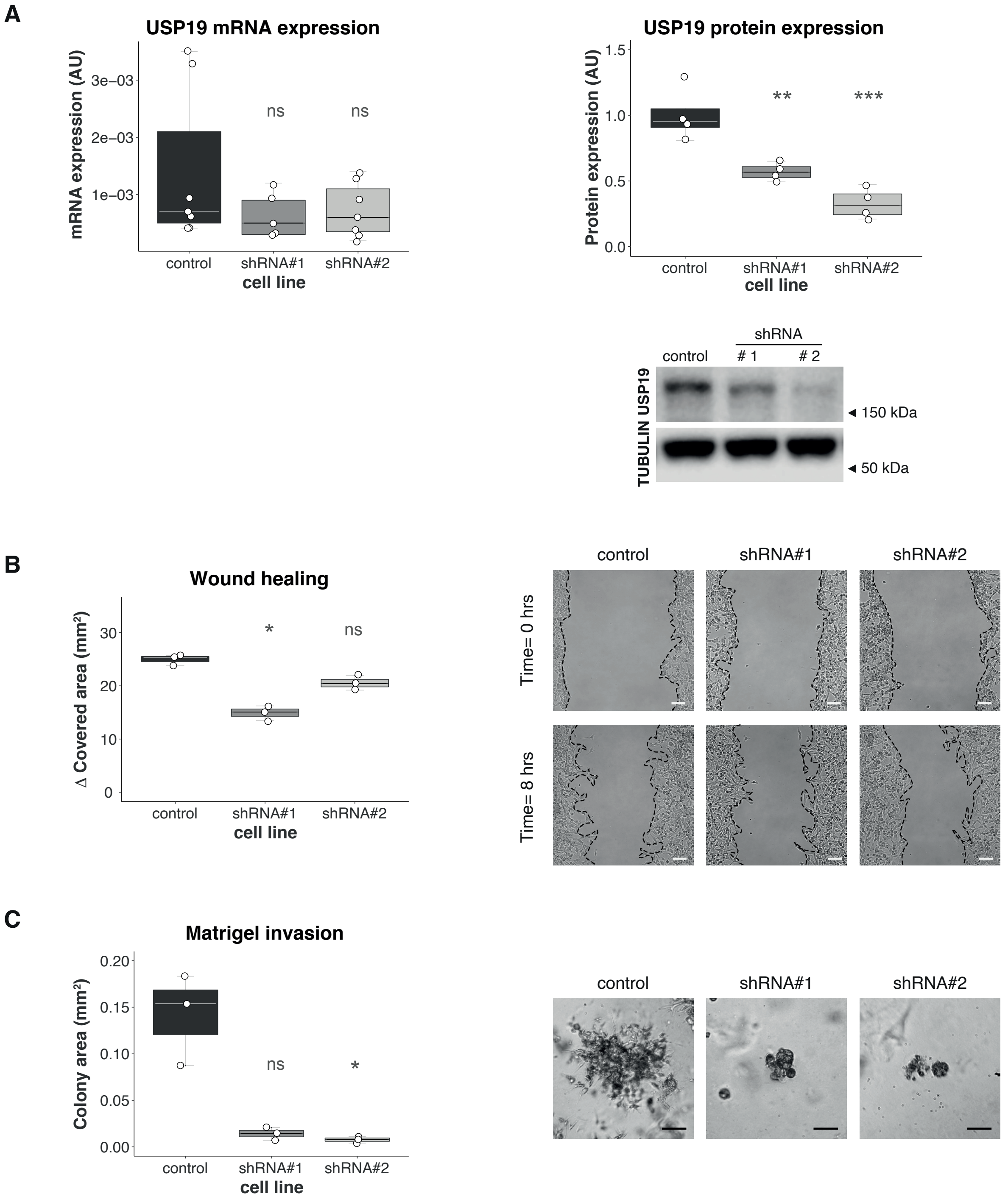

Figure S6I *In vitro* validation in MDAMB436 cells.

USP19 silencing effects were studied in MDAMB436 cells. (**A**) Left: Efficiencies of shRNA-mediated target gene knockdown were confirmed by RT-PCR (n≥ 5, Kruskal-Wallis, Dunn's multiple comparison test. shRNA#1 p= 0.6892 and shRNA#2 p= 0.8389) and Right: Western Blotting (n= 4, one-way ANOVA, Dunnett's multiple comparison test. shRNA#1 p= 0.0039 and shRNA#2 p= 0.0002). (**B**) Cells migratory potential was evaluated by wound healing assay. Left: gap covered area (mm^2) after 8 hours (n= 3, Kruskal-Wallis, Dunn's multiple comparison test. shRNA#1 p= 0.0146 and shRNA#2 p= 0.3594); right: representative areas in a wound healing experiment at the indicated time points. Scale bar= 100 µm. (**C**) Invasiveness was assessed with Matrigel 3D experiments. Left: Colony area was calculated at the end of the experiment (n= 3, Kruskal-Wallis, Dunn's multiple comparison test. shRNA#1 p= 0.2021 and shRNA#2 p= 0.0341); right: a representative area for the cell invasion assay after 5 days in culture is shown. Scale bar= 200 µm.

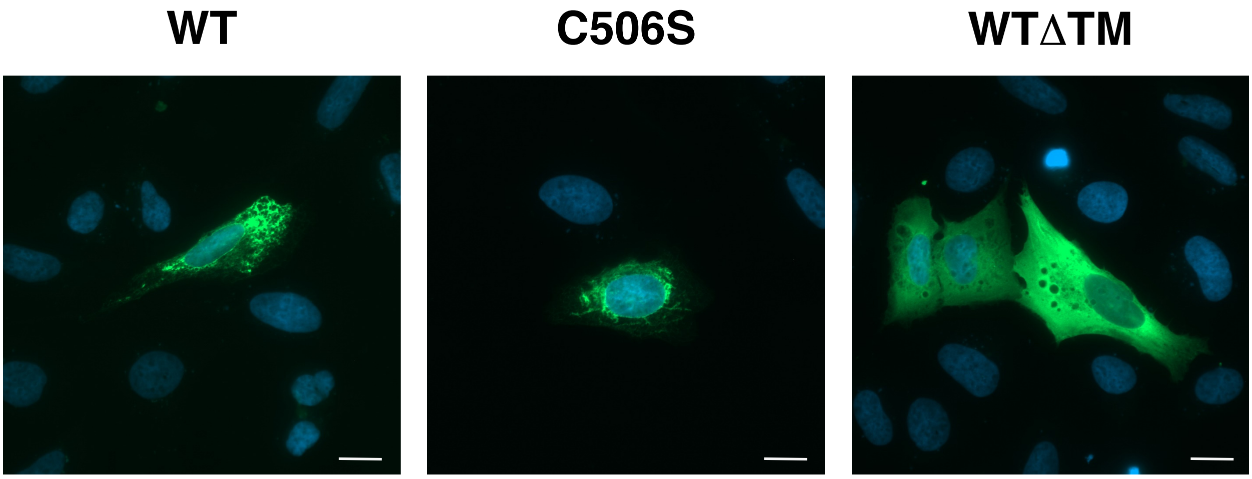

Figure S7I USP19 overexpression in U2OS cells.

There are a variety of USP19 mRNA transcripts generated by alternative splicing, and not all of them show the same functional properties. In this regard, changes in the last exon coding sequence generate soluble isoforms and endoplasmic reticulum (ER) membrane-bound isoforms. In order to analyze whether USP19's transmembrane domain was required for migration and 3D growth, we generated a GFP-tagged USP19 construct (wild type for its catalytic activity) with a point mutation that generates a premature stop codon. This mutation prevented the transmembrane domain to be translated, therefore generating a protein that mimicked USP19's soluble isoform (named WTΔTM). We analyzed overexpressed-USP19 proteins subcellular localization by transiently transfecting U2OS cells with wild type (WT), catalytically dead mutant (C506S) or cytoplasmic (WTΔTM) GFP-tagged USP19 constructs. Cells were fixed with 4% paraformaldehyde, stained with DAPI and mounted. Images were captured using an inverted fluorescence microscopy. Scale bar= 100 μm.

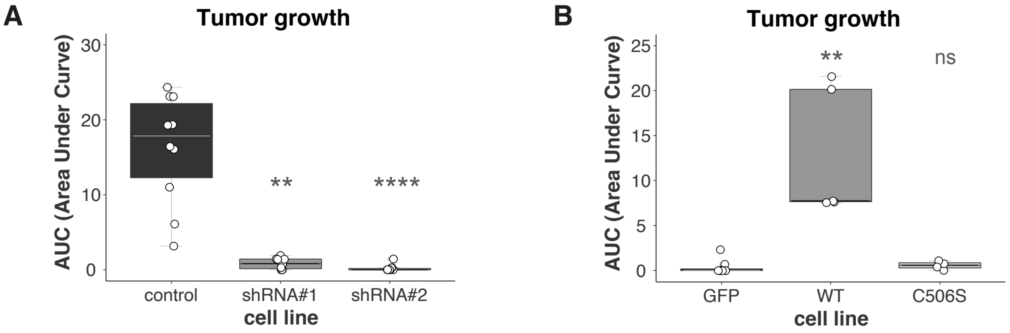

Figure S8I Tumor growth analysis.

Tumor growth was analyzed by calculating the area under the tumor volume curves of mice injected with the different cell lines, at the end of the experiment. (**A**) AUC values of mice injected with MDAMB231 cells (n≥ 10, Kruskal-Wallis, Dunn's multiple comparison test. shRNA#1 p= 0.0021 and shRNA#2 p< 0.0001). (**B**) AUC values of mice injected with MCF7 cells (n≥ 4, Kruskal-Wallis, Dunn's multiple comparison test. WT p= 0.0015 and C506S p= 0.7582).

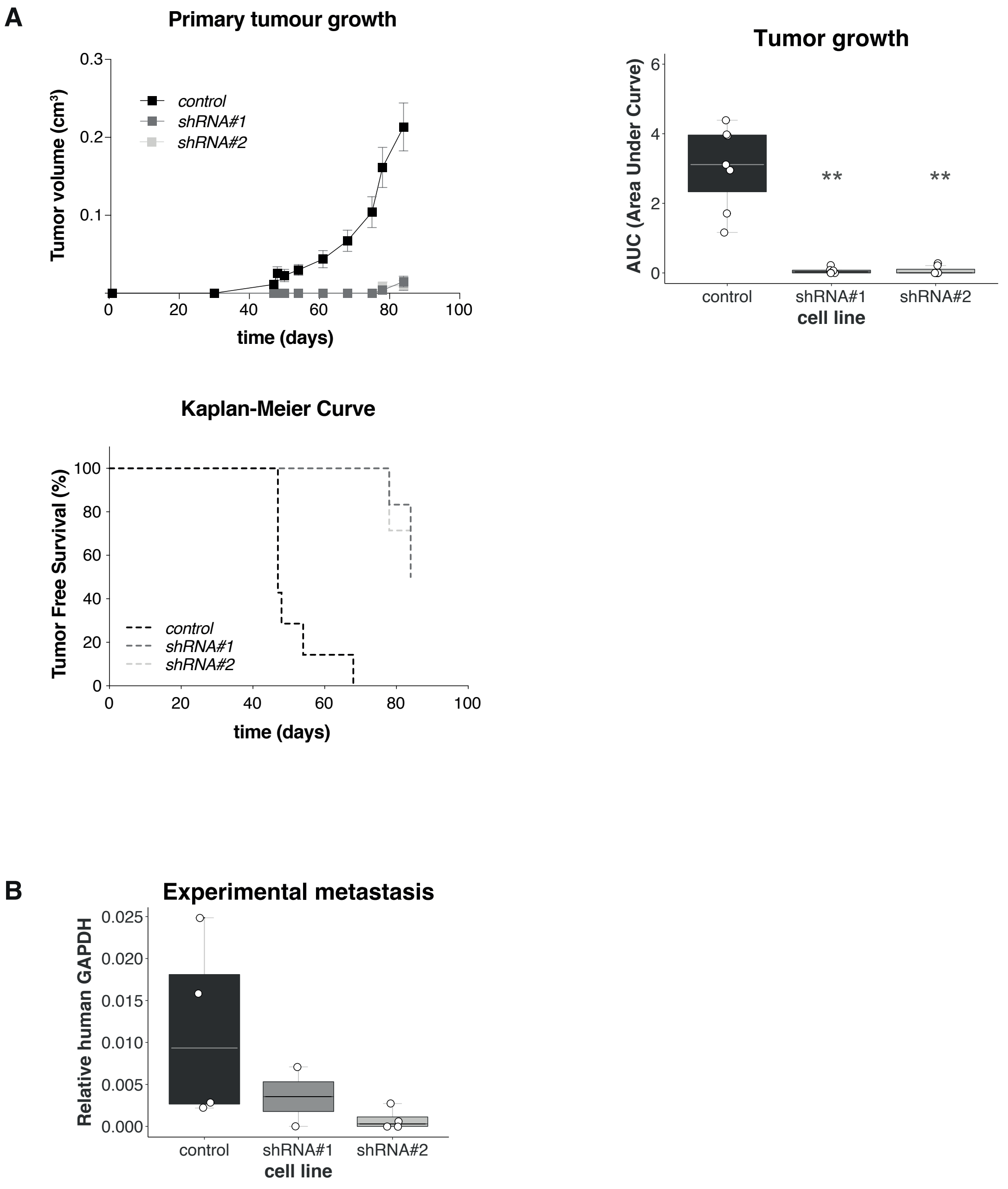

Figure S9I *In vivo* experiments with MDAMB436 cells.

(**A**) Downregulation of USP19 attenuates tumorigenicity *in vivo*: control or USP19-silenced MDAMB436 cells were subcutaneously inoculated into the mammary fat pads of female NOD/SCID mice. Top left: Tumor volume was measured at the indicated time points (results show mean value ± S.E.). Top right: the area under the tumor volume curves was calculated at the end of the experiment (n≥ 6, Kruskal-Wallis, Dunn's multiple comparison test. shRNA#1 p= 0.0053 and shRNA#2 p= 0.0014). Bottom: Kaplan-Meier curves were built for Tumor Free Survival (TFS) over time (n≥ 6, Log-Rank (Mantel-Cox) test, shRNA#1 p= 0.0003 and shRNA#2 p= 0.0001). (**B**) Experimental metastasis assay: NOD/SCID male mice were inoculated with MDAMB436 USP19-silenced cells through tail vein injection and after 2 months, lungs were harvested. Metastasis foci were estimated by qPCR human DNA quantification (n≥ 2).

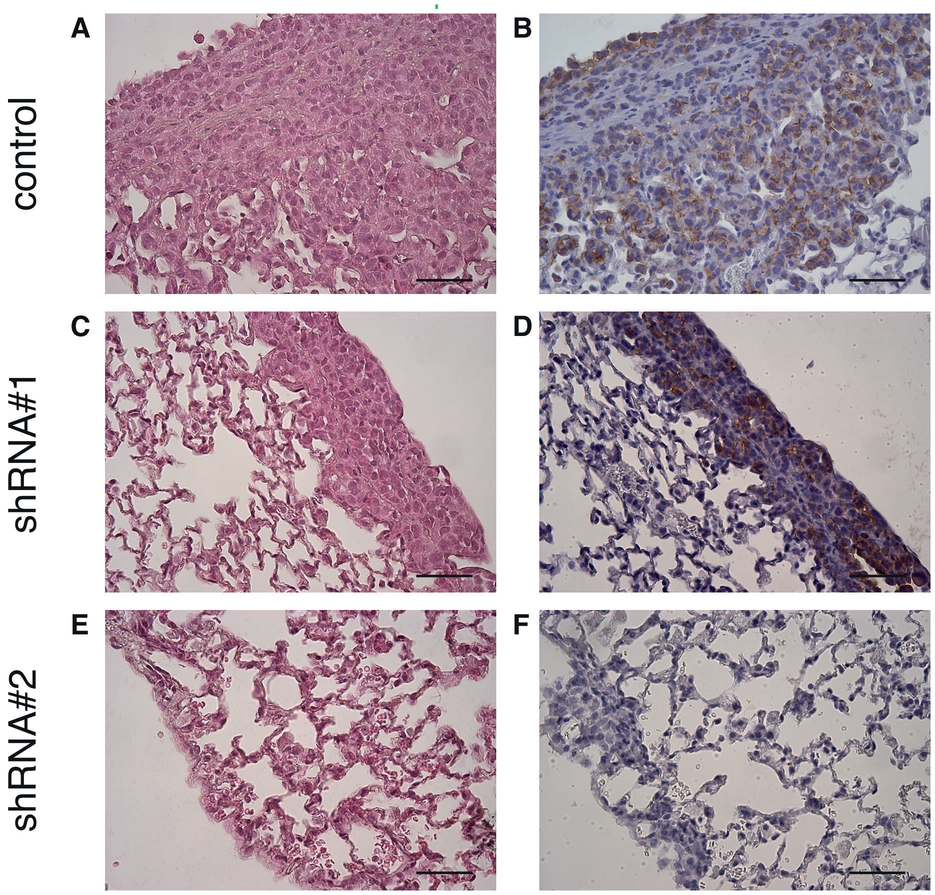

Figure S10I Metastatic load determination.

Representative images of lung sections of mice injected with control or USP19-silenced MDAMB231 cells. Tissues were analyzed for metastatic load by Hematoxylin & Eosin staining (**A**, **C**, and **E**), and nodules tumor origin was confirmed by staining with the mouse anti-human CD44s monoclonal antibody (**B**, **D**, and **F**). Scale bar: 50 µm.

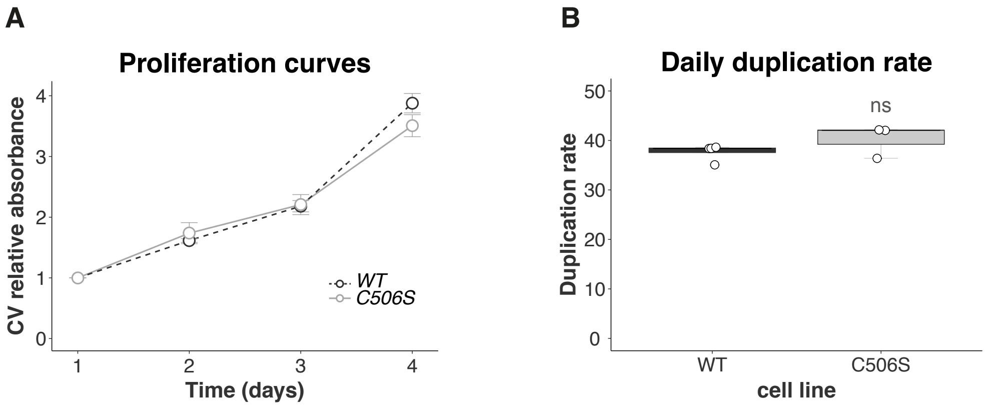

Figure S11I USP19 overexpression does not affect 2D proliferation in MCF7 cells.

Crystal violet (CV) staining was used to determine cell growth over time. Cells were seeded onto wells and allowed to attach. At the indicated time points, cells were fixed and then stained at the end of the experiment. (**A**) The graph shows the mean relative CV absorbance every 24 hours. (**B**) Doubling time was calculated for MCF7 cells overexpressing WT or mutant C506S USP19 constructs ($n \geq 3$, Mann-Whitney test, $p = 0.4000$).

A

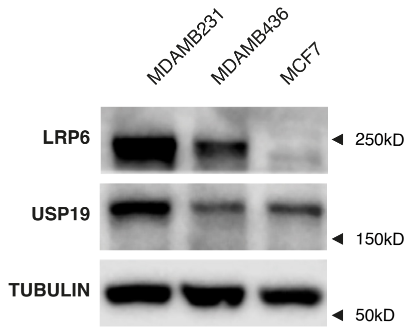

B

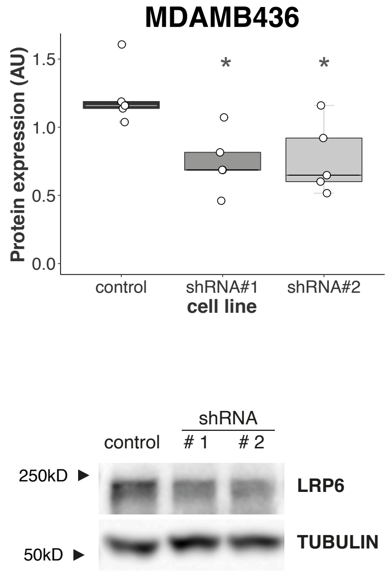

Figure S12I USP19 expression regulates LRP6 protein levels in breast cancer cell lines.

(**A**) Western blotting was performed in order to analyze LRP6 protein expression in the breast cancer cell lines used in this manuscript. (**B**) LRP6 protein expression was evaluated in control or USP19 silenced MDAMB436 cells. Top: LRP6 signal quantification (n=5, one-way ANOVA, Dunnett's multiple comparison test. shRNA#1 p= 0.0139 and shRNA#2 p= 0.0188), bottom: representative image of a blot.

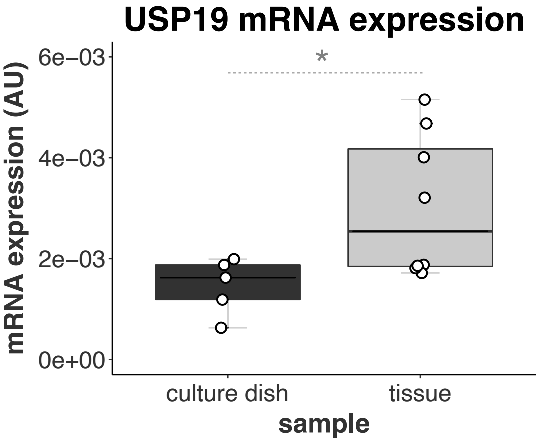

Figure S13I USP19 mRNA expression is increased in tissues.

USP19 mRNA expression levels were analyzed by RT-qPCR in cultured MDAMB231 cells expressing PLKO.1 empty vector (growing in culture dishes), or in tumors that generated in NOD/SCID mice upon injection of MDAMB231 cells expressing PLKO.1 empty vector ($n \geq 5$, Mann-Whitney test, $p = 0.0326$).

cell line		Tumor-free Median Survival (days)	Long-Rank (Mantel-Cox) test vs control		Hazard Ratio (logrank) vs control	
			p value	Summary	Ratio	95% CI
MDAMB231	control	38	-	-	-	-
	shRNA#1	64	<0.0001	****	4.315	1.347-12.96
	shRNA#2	undefined	<0.0001	****	6.845	2.149-21.80
MDAMB436	control	47	-	-	-	-
	shRNA#1	84	0.0003	***	5.768	1.468-22.67
	shRNA#2	undefined	0.0001	***	8.399	2.002-35.23
MCF7	GFP	undefined	-	-	-	-
	WT	94.5	0.0003	***	7.953	2.488-25.43
	C506S	189	0.6000	ns	1.536	0.305-7.719

Table S2I Analysis of Tumor Free Survival curves in USP19 silencing and overexpression *in vivo* experiments.

USP19 effects on tumor growth were analyzed by injecting genetically manipulated cell lines in the mammary fat pad of NOD/SCID mice and measuring tumor growth over time. For each control or USP19 silenced cell line, in the case of MDAMB231 or MDAMB436 cells, and for each control or USP19 overexpressing cell line, in the case of MCF7 cells, tumor growth curves were built. Using this data, we calculated median tumor-free survival time (expressed in days) and performed Long-Rank (Mantel-Cox) tests of each cell line relative to their corresponding control. Hazard ratio ranks relative to control treatments are also indicated in the table.

Variable	HR	95% CI	P*
**Disease-free survival**			
Tumor size, cm (>2 *vs* ≤ 2)	1.0	0.6-1.9	0.888
Tumor grade (1 *vs* 2-3)	1.1	0.5-2.8	0.793
ER (negative *vs* positive)	1.6	0.6-4.2	0.302
PR (negative *vs* positive)	1.1	0.5-2.6	0.853
Ki-67 (positive *vs* negative)	1.1	0.6-2.0	0.818
HER-2 (positive *vs* negative)	1.9	0.9-4.2	0.112
USP19 (low *vs* high)	1.0	0.6-1.9	0.926
**Distant Relapse-Free Survival**			
Tumor size, cm (≤ 2 *vs* >2)	1.4	0.6-3.1	0.413
Tumor grade (2-3 *vs* 1)	3.6	0.5-27.0	0.221
ER (negative *vs* positive)	2.7	0.9-8.8	0.090
PR (positive *vs* negative)	1.8	0.6-5.3	0.313
Ki-67 (positive *vs* negative)	1.1	0.5-2.4	0.808
HER-2 (positive *vs* negative)	1.9	0.8-4.6	0.176
USP19 (high *vs* low)	2.4	1.1-5.4	0.032*

Table S3l USP19^{High} is an independent prognostic predictor of DRFS.

Multivariate analysis of USP19 expression in breast tumors indicated that USP19^{High} is an independent prognostic predictor of DRFS. HR: Hazard Ratio, CI: Confidence Interval (n= 168, Cox's proportional hazard model, DFS p= 0.926, DRFS p= 0.032).

Variable	Value (%)
**Age at diagnosis (yr)**	
Median	56.7
<50	51 (30.4)
50-65	69 (41.1)
>65	48 (28.6)
**Menopausal status**	
Pre/perimenopausal	54 (32.1)
Postmenopausal	114 (67.9)
**Molecular subtypes**	
Luminal A	71 (42.3)
Luminal B	57 (33.9)
HER2 positive	8 (4.8)
Triple negative	32 (19.0)
**Tumor size**	
≤ 2 cm	109 (64.9)
> 2 cm	59 (35.1)
**Histotypes**	
Ductal carcinoma	135 (80.4)
Lobular carcinoma	24 (14.3)
Other	9 (5.3)
**Tumor grade**	
1	21 (12.5)
2-3	147 (87.5)
**ER**	
Negative	43 (25.6)
Positive	125 (74.4)
**PR**	
Negative	59 (35.1)
Positive	109 (64.9)
**Ki-67**	
Negative	90 (53.6)
Positive	78 (46.4)
**HER2**	
Negative	149 (88.7)
Positive	19 (11.3)
**USP19**	
Low	106 (63.1)
High	62 (36.9)
**Patient outcome**	
Without recurrence	119 (70.8)
Local recurrence	20 (11.9)
Distant recurrence	29 (17.3)

Table S5I Characterization of patient cohort used in this manuscript.

168 N0 patients with T1/T2 tumors with primary unilateral breast carcinoma from the Regina Elena National Cancer Institute, Rome, Italy were included in this study. Patients age at diagnosis, menopausal status and outcome are indicated in the table. Moreover, their tumors characteristics are stated, including molecular subtype, tumor size, histological type, grade, estrogen receptor (ER) status, progesterone receptor (PR) status, Ki-67 status, Human Epidermal Growth Factor Receptor 2 (HER2) status and USP19 staining level, as absolute and percentual values.

Variable	USP19		
	Low: n (%)	High: n (%)	P*
**Tumor size**			
≤ 2 cm	74 (69.8)	35 (56.5)	0.095
> 2 cm	32 (30.2)	27 (42.5)	
**Tumor grade**			
1	17 (16.0)	4 (6.5)	0.091
2-3	89 (84.0)	58 (93.5)	
**ER**			
Negative	27 (25.5)	16 (25.8)	1,000
Positive	79 (74.5)	46 (74.2)	
**PR**			
Negative	40 (37.7)	19 (30.6)	0.404
Positive	66 (62.3)	43 (69.4)	
**Ki-67**			
Negative	61 (57.5)	29 (46.8)	0.201
Positive	45 (42.5)	33 (53.2)	
**HER2**			
Negative	100 (94.3)	49 (79.0)	0.003*
Positive	6 (5.7)	13 (21.0)	
**Subtype**			
Luminal A	48 (67.6)	23 (32.4)	0.014*
Luminal B	31 (54.4)	26 (45.6)	
HER2	2 (25.0)	6 (75.0)	
TN	25 (63.1)	7 (36.9)	

Table S6I Characterization of USP19 protein expression in human samples.

The table shows USP19 status according to clinicopathological features of patients, including tumor size and grade, estrogen receptor (ER), progesterone receptor (PR) and Human Epidermal Growth Factor Receptor 2 (HER2) status, subtype and ki-67 staining (Pearson's χ^2 test). Cases are indicated as absolute and percentual values.

SUPPLEMENTARY MATERIALS AND METHODS

shRNA library transduction

1 mg of the shRNA library plasmid DNA was mixed with 4 mg of lentiviral packaging plasmid mix (pD8.9 and pCMV-VSVG at a 1:1 ratio) and incubated with 30 mg of polyethylenimine for 15 min at RT. The entire mixture was then added to a 100 mm dish containing Hek293T packaging cells and 48 hours after transfection, the supernatant from each dish was filtered. Target cells were transduced with a 1:27 dilution of the lentivirus stock (which ensured single shRNA infection) and were then combined at the time of harvest to reach a starting number of $4,4x10^6$ cells per condition (~300X coverage of the library complexity). 48 hours after transduction, the media was replaced with puromycin selection media and cells were then propagated for 14 days before use.

shRNA library sequencing

The library preparation strategy uses genomic DNA and two rounds of PCR in order to isolate the shRNA cassette and prepare a single strand of the hairpin for sequencing by means of an XhoI restriction digest in the stem-loop region. We used barcoded half-hairpin sequences for multiplexing purposes. The procedure was performed as described previously (1). A HiSEQ 2500 HT Mode V4 Chemistry Illumina instrument was used with a simple 1x50 run and on average $1.2x10^6$ reads were obtained per sample.

shRNA data were analyzed in a similar fashion to RNA-seq data. Briefly, quality control was performed with FastQC, reads were trimmed to include only shRNA sequences using FASTQ trimmer and filtered with the FASTQ Quality Filter. Reads were then aligned to a custom reference library of shRNA sequences using TopHat2.

Mutagenesis

Mutagenesis PCR amplification was performed with the KAPA HiFi System (Kapa Biosystems). Mutations that generated a stop codon at amino acid 1290 were corroborated by sequencing and by checking expression in U2OS cells under a fluorescence microscopy (Supp. Fig. 7). Mutagenesis primers sequences are as follows:

5'-GATGAGGGCTGCCTCCGGTAGTAGTAGCTGGGCACCGTGGCGG-3',

5'-CCGCCACGGTGCCCAGCTACTACTACCGGAGGCAGCCCTCATC-3'.

Primers list

For mRNA expression analysis:

Primer name	Primer sequence
Usp19 sense	5′-*CAAATGTTCTCATCGTGCAGCTC*-3′
Usp19 antisense	5′-*CTTGCTCAGGTCCAGGTTCCTAACA*-3′
GAPDH sense	5’-*TGCACCACCAACTGCTTAGC*-3′
GAPDH antisense	5′-*GGCATGGACTGTGGTCATGAG*-3′

For DNA expression analysis:

Primer name	Primer sequence
Human GAPDH sense	5′-*TACTAGCGGTTTTACGGGCG*-3′
Human GAPDH antisense	5′-*TCGAACAGGAGGAGCAGAGAGCGA*-3′
mouse GAPDH sense	5’- *CCTGGCGATGGCTCGCACTT* -3′
mouse GAPDH antisense	5′- *ATGCCACCGACCCCGAGGAA* -3′

Crystal violet proliferation assay

$1x10^4$ cells were seeded in 96-well plates at time= 0 hours in quadruple. Every 24 hours, medium was removed, cells were rinsed with 1X PBS and fixed with methanol. After 4 days, wells were stained with 150 µl of a 0.05% crystal violet solution. Plates were then rinsed with water, dried out and the remaining crystal violet was solubilized in 150 µl methanol. The absorbance was measured at 595 nm.

Area-based analysis of proliferation rate

This experiment was performed as described previously (2), with minor modifications. Briefly, $1x10^3$ cells were seeded in 96-well plates and incubated overnight to allow cell attachment. Images were then acquired under bright field illumination every 6 hours for 3 days using a Zeiss Axio Observer Z1 Inverted Epi-fluorescence microscope equipped with an AxioCam HRm3digital CCD camera, a Stage Controller XY-STEP-SMC-2009 scanning stage and an Incubator XLmulti-S1 and Heating Unit XL-S1 for live imaging incubation. A 10X air objective and Zeiss Zen Blue 2011 software was used for image acquisition. Image analysis was performed with Fiji software, using an automated analysis macro to measure the occupied area by cells.

Wound healing assays

For wound-edge cells analysis, GFP-tagged H2B expressing MDAMB231 cells were used. Images were acquired every 30 minutes for 8 hours for three independent experiments, and calculations were performed using cell tracks from each wound edge separately. The number and position of cells were

determined using, ImageJ/Fiji -'Trackmate' plug-in (3), and trajectory analysis was performed using the 'Chemotaxis and Migration Tool' 1.01 ImageJ plug-in (Ibidi).

Agar invasion assay

5 µL spots of a 1% noble agar melted solution were pipetted onto 96 well cell culture plates and allowed to cool for 20 min at RT. At this point, 5x103 cells were plated in culture media supplemented with 1 µg/ml Hoechst 33258 (Thermo Fisher Scientific) and allowed to adhere for 1 hour. Fluorescent images of the edges of each spot were taken every 20 minutes for 18 hours using a 10X magnification air objective, using the Fluorescent Microscope mentioned before. The number and position of cells were determined using ImageJ/Fiji -'Trackmate' plug-in (3).

Noble agar assay

A 2 ml mixture of 5,000 cells in assay medium and 0.3% noble agar was seeded onto a 4 ml-solidified bed of 0.6% noble agar on six-well plates. The plates were incubated at 37°C. and the cultures were fed once a week with assay medium, for 6 weeks.

Matrigel three-dimensional cell culture

1,000 cells were cultured embedded in 100 µl basement membrane gels composed of 9.2 µg/µl Matrigel (BD Bioscience, Cat#356231) in 96 well plates. 50 µl of fresh medium were added on top of each gel every three days.

Metastatic load quantification of lung Hematoxylin & Eosin (H&E) stained slides

The presence of tumor nodules was identified by scanning individual lung H&E-stained slides with an optical microscope. Digital image files were acquired for each specimen, and Adobe Photoshop software was used to determine the percentage of the lung section that was occupied by the tumor. Immunohistochemical staining with the anti-human CD44s (HCAM) antibody (clone DF1485; heat induced epitope retrieval in citrate buffer pH 6.0; 1:50, incubation time: 60') was used to confirm the tumor origin of the lung nodules (Supp. Fig. 10).

Patients

We retrospectively extracted the eligible patients for the study from a consecutive cohort of cases (year range, 1983-2001) diagnosed with primary unilateral breast carcinoma at the Regina Elena National Cancer Institute, Rome, Italy. From the original series, only N0 patients with T1/T2 tumors were included in this study (n= 168). The patient and tumor characteristics can be found in Supplementary Tables 5 and 6. This study was reviewed and approved by the Ethics Committee of the Regina Elena National Cancer Institute, and written informed consent was obtained from all patients. All the patients were treated with quadrantectomy and received radiation therapy, while 90

(53,6%) received chemotherapy associated or not to hormonal therapy, and 58 (34.5%) underwent only hormonal therapy. Patients with HER2-positive tumors did not receive trastuzumab because it was not available during the study period. The median follow-up was 91.5 months (range, 6 - 298 months). Follow-up data were collected from institutional records or referring physicians. During the follow-up, distant relapse was seen in 29 (17.3%) of the patients.

References

1. Sullivan K.D., Lewis H.C., Hill A.A., Pandey A., Jackson L.P., Cabral J.M., Smith K.P., Liggett L.A., Gomez E.B., Galbraith M.D., DeGregori J., Espinosa J.M. Trisomy 21 consistently activates the interferon response. eLife. 5: (2016). doi: 10.7554/eLife.16220

2. Bahnson A., Athanassiou C., Koebler D., Qian L., Shun T., Shields D., Yu H., Wang H., Goff J., Cheng T., Houck R., Cowsert L. Automated measurement of cell motility and proliferation. BMC Cell Biol. 6(1): 19 (2005). doi: 10.1186/1471-2121-6-19

3. Tinevez J.Y., Perry N., Schindelin J., Hoopes G.M., Reynolds G.D., Laplantine E., Bednarek S.Y., Shorte S.L., Eliceiri K.W. TrackMate: An open and extensible platform for single-particle tracking. Methods. 115: 80-90 (2017). doi: 10.1016/j.ymeth.2016.09.016
